# TNIP3 protects against pathological cardiac hypertrophy by stabilizing STAT1

**DOI:** 10.1038/s41419-024-06805-4

**Published:** 2024-06-26

**Authors:** Hongjie Shi, Yongjie Yu, Dajun Li, Kun Zhu, Xu Cheng, Tengfei Ma, Zhangqian Tao, Ying Hong, Zhen Liu, Siyi Zhou, Jianqing Zhang, Yun Chen, Xiao-Jing Zhang, Peng Zhang, Hongliang Li

**Affiliations:** 1 Taikang Medical School (School of Basic Medical Sciences), Wuhan University, 430000 Wuhan, China; 2 Institute of Model Animal, Wuhan University, 430000 Wuhan, China; 3 Gannan Innovation and Translational Medicine Research Institute, Gannan Medical University, 341000 Ganzhou, China; 4 State Key Laboratory of New Drug Discovery and Development for Major Diseases, Gannan Innovation and Translational Medicine Research Institute, 341000 Ganzhou, China; 5 Department of Neurosurgery, Huanggang Central Hospital, 438000 Huanggang, China; 6 Huanggang Institute of Translational Medicine, 438000 Huanggang, China; 7 Department of Cardiology, Renmin Hospital of Wuhan University, 430000 Wuhan, China; 8 Clinical trial centers, Huanggang Central Hospital, 438000 Huanggang, China

**Keywords:** Cardiac hypertrophy, Heart failure

## Abstract

Pathological cardiac hypertrophy is one of the major risk factors of heart failure and other cardiovascular diseases. However, the mechanisms underlying pathological cardiac hypertrophy remain largely unknown. Here, we identified the first evidence that TNFAIP3 interacting protein 3 (TNIP3) was a negative regulator of pathological cardiac hypertrophy. We observed a significant upregulation of TNIP3 in mouse hearts subjected to transverse aortic constriction (TAC) surgery and in primary neonatal rat cardiomyocytes stimulated by phenylephrine (PE). In *Tnip3*-deficient mice, cardiac hypertrophy was aggravated after TAC surgery. Conversely, cardiac-specific *Tnip3* transgenic (TG) mice showed a notable reversal of the same phenotype. Accordingly, TNIP3 alleviated PE-induced cardiomyocyte enlargement in vitro. Mechanistically, RNA-sequencing and interactome analysis were combined to identify the signal transducer and activator of transcription 1 (STAT1) as a potential target to clarify the molecular mechanism of TNIP3 in pathological cardiac hypertrophy. Via immunoprecipitation and Glutathione S-transferase assay, we found that TNIP3 could interact with STAT1 directly and suppress its degradation by suppressing K48-type ubiquitination in response to hypertrophic stimulation. Remarkably, preservation effect of TNIP3 on cardiac hypertrophy was blocked by STAT1 inhibitor Fludaradbine or STAT1 knockdown. Our study found that TNIP3 serves as a novel suppressor of pathological cardiac hypertrophy by promoting STAT1 stability, which suggests that TNIP3 could be a promising therapeutic target of pathological cardiac hypertrophy and heart failure.

## Introduction

Heart disease continues to cause the highest rates of morbidity and mortality globally [1, 2]. Cardiac hypertrophy is a common process in the development of heart failure, involving compensatory thickening of the muscle in response to stressors such as mechanical pressure, growth factors, cytokines, catecholamines, and genetic predisposition. However, persistent response could lead to the development of pathological cardiac hypertrophy, which has been identified as an independent predictor of adverse cardiovascular outcomes, including myocardial infarction, arrhythmia, and heart failure [3–5]. Numerous basic studies indicated that myocardial hypertrophy is a complex pathological process involving numerous genes and signaling pathways [6–10]. However, there is still a lack of effective strategies to alleviate pathological cardiac hypertrophy in clinical trials. Therefore, finding new regulating targets for pathological cardiac hypertrophy will supply new therapeutic strategies.

TNFAIP3 interacting protein 3 (TNIP3), also known as ABIN3, was initially identified in human monocyte-like macrophages during infection with Listeria monocytogenes [11]. TNIP3 has been shown to negatively regulate NF-κB activation in response to LPS [12], and has also been implicated in inflammatory bowel disease [13]. Focusing on research in cardiovascular metabolic diseases for several years, our previous studies have demonstrated that TNIP3 acts as a novel endogenous suppressor of nonalcoholic steatohepatitis by interrupting TAK1 activation [14]. Furthermore, TNIP3 overexpression has shown partial protective effects against hepatic ischemia/reperfusion injury through the degradation of LATS2 [15]. Metabolic disorders play a significant role in triggering cardiovascular diseases. However, the role of TNIP3 in cardiovascular diseases, especially cardiac hypertrophy, has not been reported, highlighting a current gap in our understanding of this area.

In present study, our findings revealed a significant upregulation of TNIP3 expression in cardiac hypertrophy. Notably, the absence of TNIP3 led to enhanced cardiomyocyte hypertrophy and cardiac interstitial fibrosis in mouse model of cardiac hypertrophy induced by transverse aortic constriction (TAC). Conversely, cardiac-specific *Tnip3* transgenic mitigated the progression of pathological cardiac hypertrophy in mice. Furthermore, our detection revealed an interaction between TNIP3 and signal transducer and activator of transcription 1 (STAT1), which promoted the stability of STAT1 by inhibiting its K48-type ubiquitination in response to hypertrophic stimulation.

## Results

### TNIP3 is upregulated in pathological cardiac hypertrophy

**Fig. 1:**
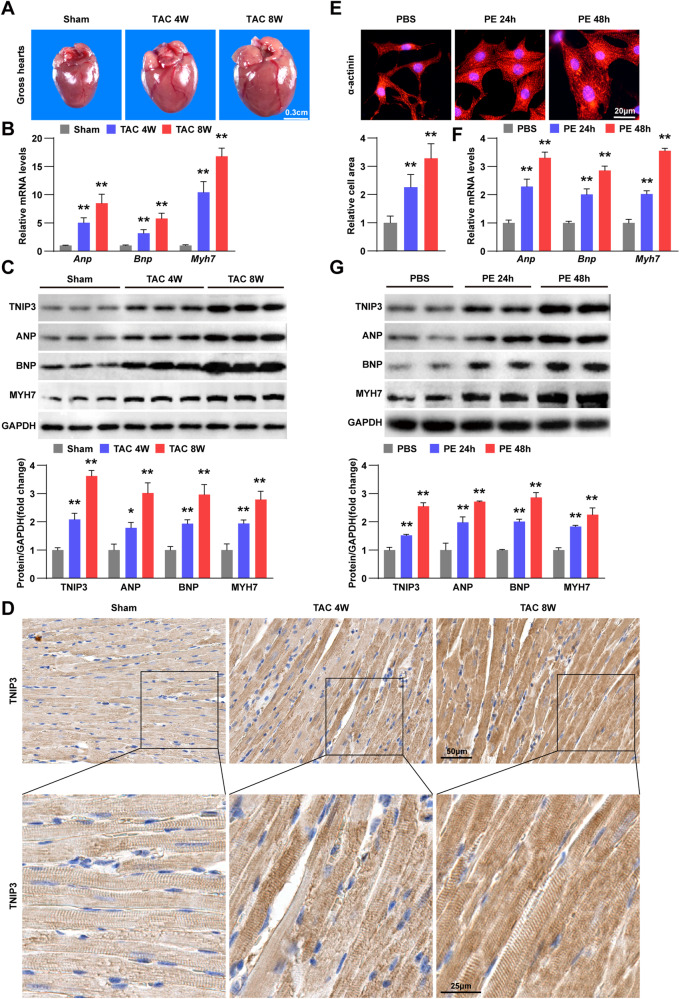
TNIP3 is promoted in cardiac hypertrophy model. **A** Representative images of gross hearts in mice subjected to sham or TAC surgery at indicated time point (*n* = 6). Scale bars, 0.3 cm. **B** Relative mRNA expression of hypertrophic marker genes in hearts of mice of indicated groups (*n* = 6). **C** Representative western blots (upper) and quantification (bottom) of TNIP3, ANP, BNP and MYH7 expression in heart tissues in each group (*n* = 3). **D** Representative images of TNIP3 immunohistochemical staining from indicated groups (*n* = 3). **E** Representative images of α-actinin immunofluorescence staining (upper) and quantification (bottom) in NRCMs treated with PBS or PE at indicated time point. Scale bars, 20 μm. **F** RT-PCR analysis of hypertrophic marker genes expression of indicated groups (*n* = 5). **G** Representative western blots (upper) and quantification (bottom) of TNIP3, ANP, BNP and MYH7 expression from indicated groups (*n* = 3). **P < 0.01 vs Sham or PBS group. Statistical analysis was carried out by one-way ANOVA.

To investigate whether TNIP3 participated in regulation of pathological cardiac hypertrophy, we successfully conducted the model of cardiac hypertrophy in mice subjected to TAC surgery (Fig. 1A). Real-time quantitative PCR revealed a substantial increase in mRNA levels of atrial natriuretic peptide (*Anp*), b-type natriuretic peptide (*Bnp*) and β-myosin heavy chain(*β-Mhc/Myh7*) in hypertrophic mouse hearts (Fig. 1B). Significantly elevated protein expression of TNIP3, ANP, BNP and MYH7 were observed in the cardiac hypertrophic mouse model (Fig. 1C). To further conform the localization of TNIP3 in the heart, we performed immunohistochemistry staining and observed that the upregulation of TNIP3 mainly occurred in cardiomyocytes **(**Fig. 1D). Subsequently, we established a hypertrophic model in vitro using primary neonatal rat cardiomyocytes (NRCMs) stimulated by phenylephrine (PE) (Fig. 1E–G). Consistent with the observations in mice, TNIP3 exhibited significant upregulation in PE-treated NRCMs (Fig. 1G). Overall, these results showed that TNIP3 was upregulated in vivo and in vitro in response to pressure overload, indicating that TNIP3 might be involved in the development of pathological cardiac hypertrophy.

### TNIP3 deficiency exacerbates cardiac dysfunction after TAC-induced pathological cardiac hypertrophy

**Fig. 2:**
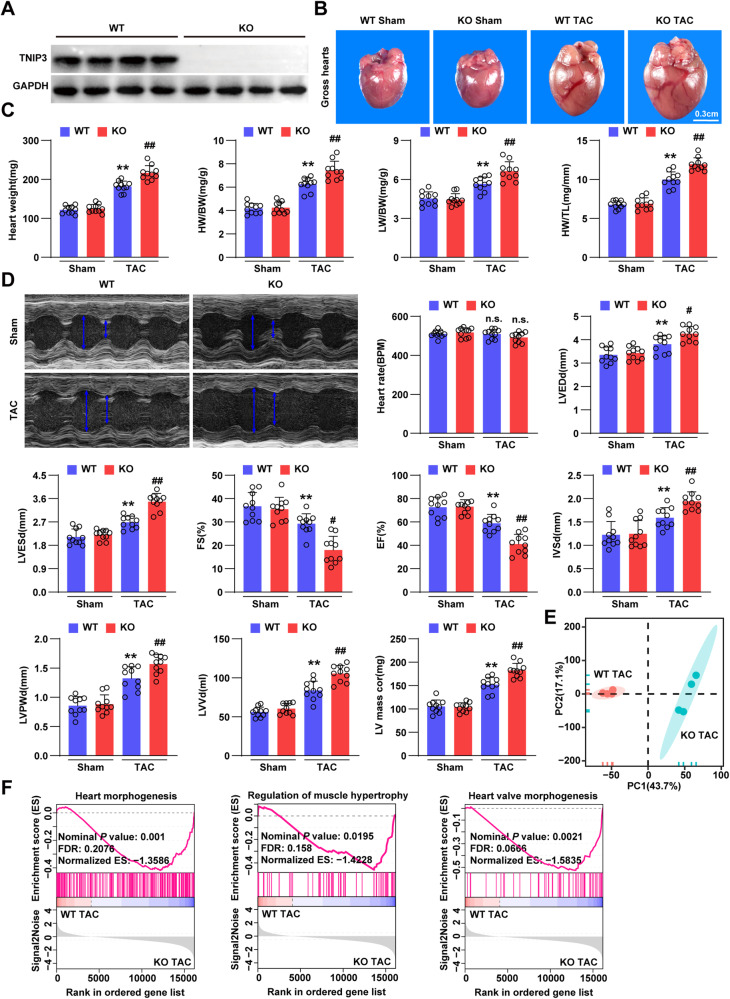
TNIP3 deletion exacerbates TAC–induced cardiac dysfunction in vivo. **A** Western blotting analysis of TNIP3 expression in WT and KO mice hearts (*n* = 4). **B** Representative images of gross hearts in WT and KO mice after sham or TAC surgery for 4 weeks (*n* = 6). Scale bars, 0.3 cm. **C** Heart weight (left), HW/BW (middle), LW/BW (middle), and HW/TL (right) ratios of each group (*n* = 10). **D** Representative echocardiography images and parameters for HR, LVEDd, LVESd, FS, EF, IVSd, LVPWd, LVVd and LV mass cor of indicated groups (*n* = 10). **E** PCA of sample distribution profiles based on the RNA-seq from indicated groups (*n* = 4). **F** GSEA showed the majority enriched genes involved in heart function according to transcriptomics data in mice hearts from indicated groups. ^n.s.^*P* ≥ 0.05 or **P < 0.01 vs WT Sham group, ^n.s.^*P* ≥ 0.05 or ^#^*P* < 0.05 or ^##^*P* < 0.01 vs WT TAC group. Statistical analysis was carried out by one-way ANOVA.

To clarify the role of TNIP3 in pathological cardiac hypertrophy, we constructed global *Tnip3* knockout (KO) mice and the deficiency of TNIP3 was identified by western blot analysis (Fig. 2A). Then *Tnip3* KO mice with their wild type (WT) littermate controls were subjected to a sustained chronic pressure overload model via TAC surgery. No significant differences were observed in the gross morphology of the heart, heart weight (HW), heart weight to body weight ratio (HW/BW), lung weight (LW)/BW, HW/tibia length (TL) or echocardiographic parameters between *Tnip3* KO and WT mice following sham surgery (Fig. 2B–D). In contrast, *Tnip3* KO mice displayed a significant increase in gross morphological parameters, including heart size, HW, HW/BW, LW/BW, and HW/TL, after four weeks of TAC surgery compared to the WT TAC group (Fig. 2B, C). The deterioration in heart function was observed in *Tnip3* KO TAC mice compared to WT TAC mice. Specifically, *Tnip3* KO TAC mice exhibited increases in several cardiac parameters, including left ventricular (LV) end-diastolic diameter (LVEDd), LV end-systolic diameter (LVESd), intra-ventricular septum diastole (IVSd), LV posterior wall thickness at end diastole (LVPWd), LV volume in diastole (LVVd), and corrected LV mass (LV mass cor). Additionally, there was a decrease in fractional shortening (FS) and ejection fraction (EF) in *Tnip3* KO TAC mice. Notably, no significant change was observed in heart rate (HR) (Fig. 2D). To investigated the effect of TNIP3 at molecular level, we conducted RNA-sequencing analysis of KO and WT hearts induced by TAC. The transcriptomic profiles result showed that KO TAC and WT TAC groups were obviously separated into two clusters (Fig. 2E). Gene set enrichment analysis (GSEA) revealed enrichment of genes associated with heart morphogenesis, regulation of muscle hypertrophy, and heart valve morphogenesis related to cardiac function in TNIP3-deficient mice following TAC surgery (Fig. 2F). Thus, TNIP3 deficiency promoted cardiac dysfunction in pathological cardiac hypertrophy after pressure overload.

### TNIP3 deletion aggravates cardiac remodeling after TAC-induced pathological cardiac hypertrophy

**Fig. 3:**
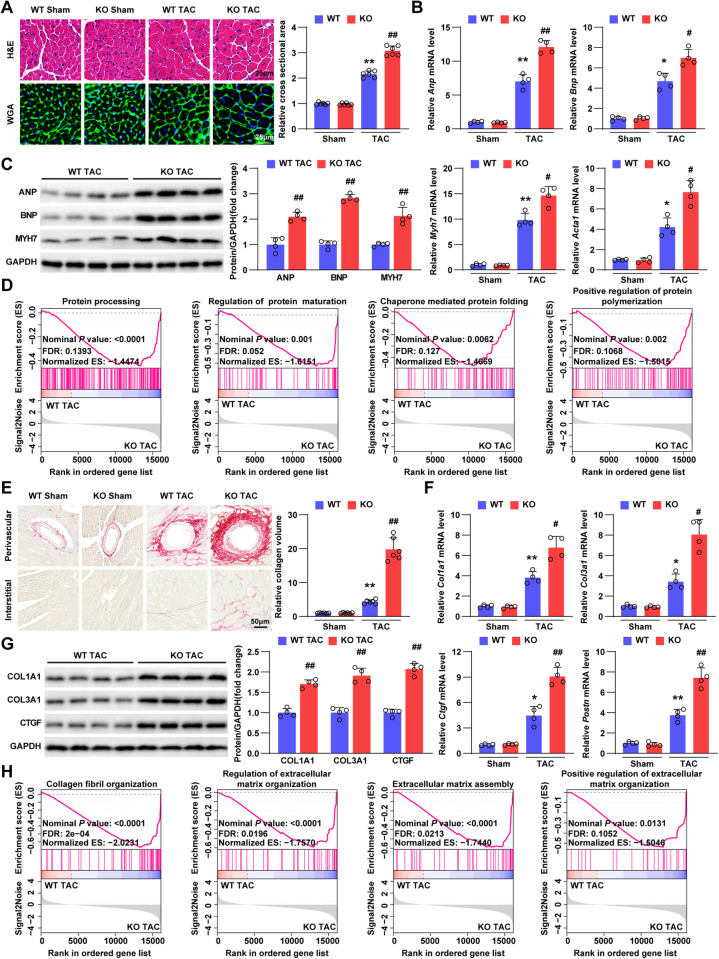
TNIP3 (TNFAIP3 interacting protein 3) deletion aggravates transverse aortic constriction (TAC)-induced cardiac remodeling in vivo. **A** Representative H&E (upper) and WGA (bottom) stained heart tissues of WT and KO mice after 4 weeks of sham or TAC surgery (*n* = 6). Scale bars, 25 μm. **B** Relative mRNA expression of hypertrophic marker genes in heart tissues from indicated groups (*n* = 4). **C** Representative western blots (left) images and quantification (right) of hypertrophic markers in each group (*n* = 4). **D** GSEA showed the majority enriched genes involved in protein processing according to RNA-seq data in hearts from indicated groups. **E** Histological analyses of the PSR staining (left) and quantification (right) of LV interstitial collagen volume of each group (*n* = 6). Scale bars, 50μm. **F** The relative mRNA expression of fibrosis marker genes in each group (*n* = 4). **G** Representative western blots (left) images and quantification (right) of fibrosis marker in each group (*n* = 4). **H** GSEA showed the majority enriched genes involved in fibrosis according to RNA-seq data in hearts from indicated groups. *P < 0.05 or **P < 0.01 vs WT Sham group, ^#^*P* < 0.05 or ^##^*P* < 0.01 vs WT TAC group. Statistical analysis was carried out by one-way ANOVA and Two-tailed Student’s t-test.

Cardiac remodeling was essential in pathological cardiac hypertrophy, therefore we further explored whether TNIP3 could regulate this process in hypertrophic mice hearts. In the setting of pathological hypertrophy, TNIP3 deficiency led to a significant increase in the cross-sectional area of cardiomyocytes (Fig. 3A). Corroborating the alterations in cardiomyocyte area, the expression of hypertrophic marker genes (*Anp*, *Bnp*, *Myh7*, alpha-actin [*Acta1*]) and corresponding proteins (ANP, BNP, MYH7), along with protein processing-related genes, showed significant upregulation and enrichment in the KO TAC group (Fig. 3B–D). However, there were no significant differences observed between the sham groups (Fig. S3A). Meanwhile, KO mice exhibited larger areas of fibrosis in hearts than WT mice after TAC surgery for four weeks (Fig. 3E). Consistently, the fibrotic related genes (collagen type I alpha 1 [*Col1a1*], collagen type III alpha 1[*Col3a1*], connective tissue growth factor [*Ctgf*], periostin [*Postn*]) and proteins (COL1A1, COL3A1, CTGF) expression were significantly upregulated in the hypertrophic hearts of KO mice (Fig. 3F, G), but with no change between sham groups (Fig. S3B). Additionally, GSEA of RNA-sequencing data demonstrated much more enrichment of fibrosis-related genes in the KO TAC group (Fig. 3H). Taken together, these results indicated that TNIP3 deficiency exacerbated cardiac hypertrophy induced by pressure overload.

### Cardiac-specific overexpression of TNIP3 alleviates pathological cardiac hypertrophy induced by pressure overload

**Fig. 4:**
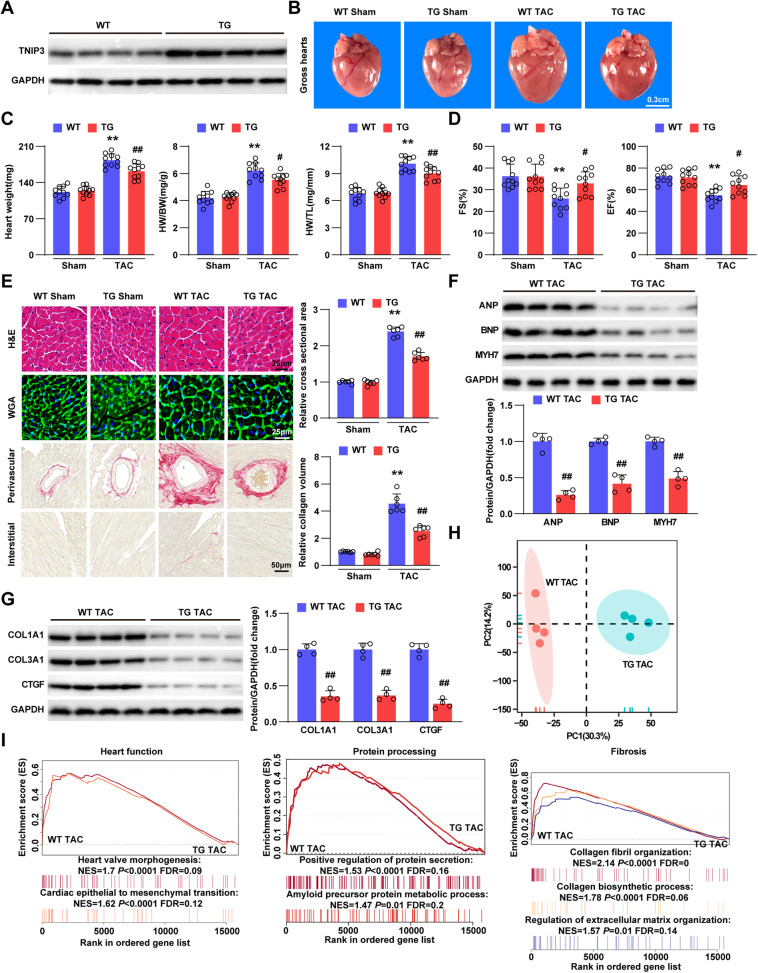
Cardiac-specific overexpression of TNIP3 attenuates pathological cardiac hypertrophy. **A** Western blotting analysis of TNIP3 protein expression in heart tissues from TG and control mice (*n* = 4). **B**, Representative images of gross hearts in WT and TG mice after sham or TAC surgery for 4 weeks (*n* = 6). Scale bars, 0.3 cm. **C**, Heart weight (left), HW/BW (middle), and HW/TL (right) ratios in each group (*n* = 10). **D**, Echocardiography parameters for FS and EF in each group (*n* = 10). **E** Representative H&E (upper, Scale bars, 25 μm), WGA (middle, Scale bars, 25 μm) and PSR (bottom, Scale bars, 50μm.) staining and quantification (right) in each group (*n* = 6). **F**, **G** Representative western blots images and quantification of hypertrophic markers and fibrotic markers in each group (*n* = 4). **H** PCA of sample distribution profiles based on RNA-seq from indicated groups (*n* = 4). **I**, GSEA showed the majority enriched genes involved in heart function, protein processing and fibrosis from indicated groups. **P < 0.01 vs WT Sham group, ^#^*P* < 0.05 or ^##^*P* < 0.01 vs WT TAC group. Statistical analysis was carried out by one-way ANOVA and Two-tailed Student’s t-test.

To further explore the influence of TNIP3 in cardiomyocytes on cardiac hypertrophy, we generated cardiomyocyte-specific *Tnip3* transgenic (TG) mice by *α-MHC* promoter. Then overexpression of TNIP3 in the mouse heart was detected by western blotting (Fig. 4A). Conversely to *Tnip3* KO mice, *Tnip3* TG mice exhibited markedly reduced heart size, lower heart weight, HW/BW, HW/TL, and LW/BW compared to WT controls following four weeks of TAC stimulation (Figs. 4B, C, S1A). Overexpression of TNIP3 mitigated pressure overload-induced cardiac dysfunction (Figs. 4D, S1B). Compared with WT mice, TNIP3 overexpression decreased cardiomyocytes enlargement and collagen deposition in response to pressure overload (Fig. 4E). Mice with cardiac-specific overexpression of TNIP3 also suppressed mRNA and protein level of cardiac hypertrophy and fibrosis marker (Figs. 4F, G, S1C, D). Furthermore, the impact of TNIP3 on heart was validated through RNA-sequencing profiles, which revealed distinct clustering (Fig. 4H), with less enrichment of heart function, protein processing, and fibrosis-related genes in TG mice hearts subjected to TAC surgery compared to WT TAC mice (Fig. 4I). Therefore, TNIP3 overexpression relieved TAC induced pathological cardiac hypertrophy.

### TNIP3 ameliorates phenylephrine induced cardiomyocyte hypertrophy in vitro

**Fig. 5:**
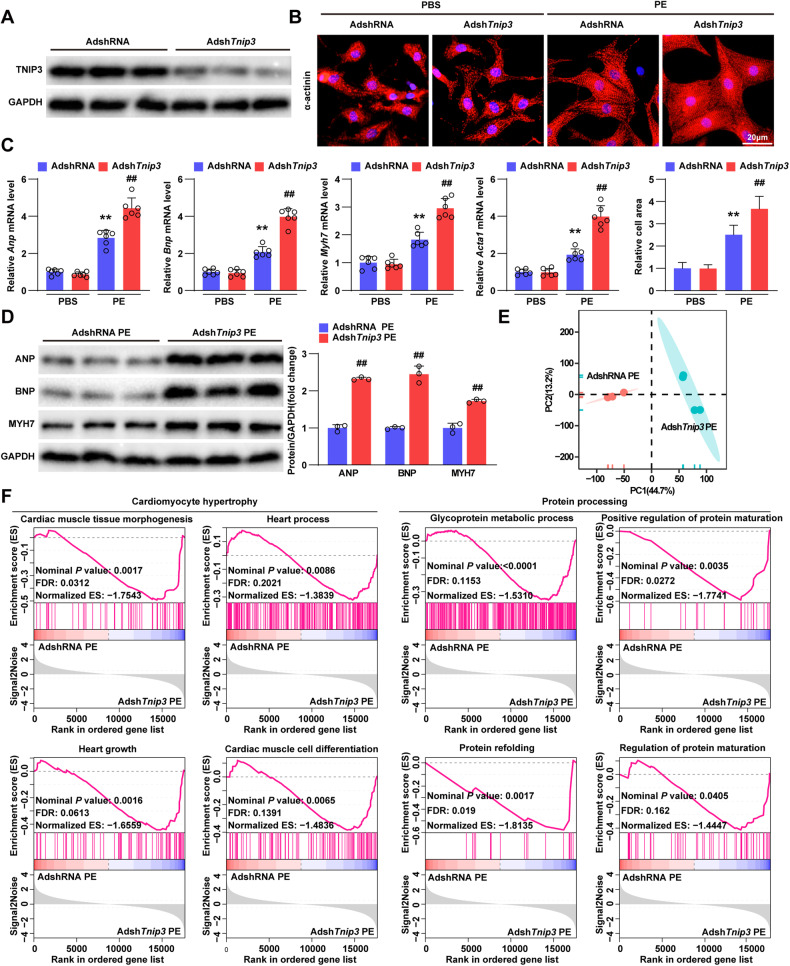
TNIP3 knockdown aggravates phenylephrine induced cardiomyocyte hypertrophy. **A** Representative Western blots of TNIP3 in NRCMs infected by Adsh*Tnip3* (*n* = 3). **B** Representative images of α-actinin immunofluorescence staining (upper) and quantification (bottom) in Adsh*Tnip3* and AdshRNA-infected NRCMs treated with PBS or PE (50 μM) for 24 h (*n* > 50 cells). Scale bars, 20 μm. **C** Relative mRNA expressions of hypertrophic marker genes in each group (*n* = 6). **D** Representative western blots (left) images and quantification (right) of hypertrophic markers from indicated groups. **E** PCA of sample distribution profiles from indicated groups based on RNA-seq (*n* = 4). **F** GSEA showed the majority enriched genes involved in cardiomyocyte hypertrophy and protein processing from indicated groups. **P < 0.01 vs AdshRNA PBS group, ^##^*P* < 0.01 vs AdshRNA PE group. Statistical analysis was carried out by one-way ANOVA and Two-tailed Student’s t-test.

To demonstrate the role of TNIP3 directly in cardiomyocytes, adenoviral short hairpin RNA targeting *Tnip3* (Adsh*Tnip3*) was constructed to knockdown TNIP3 in primary cardiomyocytes (Fig. 5A). Immunofluorescent staining for α-actinin showed that TNIP3 knockdown increased the size of cardiomyocytes following PE stimulation for 24 h (Fig. 5B), accompanied by significantly increased mRNA and protein levels of hypertrophic markers (Fig. 5C, D). The transcriptomic profiles between the Adsh*Tnip3* PE and adenoviral short hairpin RNA empty control (AdshRNA) PE groups were classified into two clusters (Fig. 5E). Moreover, GSEA revealed that TNIP3 knockdown promoted the enrichment of genes related to cardiomyocyte hypertrophy and protein processing (Fig. 5F). Thus, consistent with findings in vivo, downregulation of TNIP3 aggravated PE induced cardiomyocyte hypertrophy in vitro.

To further confirm the function of TNIP3, we increased the expression of TNIP3 in cardiomyocytes by infecting with adenoviral expressing *Tnip3* (Ad*Tnip3*) (Fig. S2A).

Overexpression of TNIP3 significantly reduced PE-induced enlargement of cardiomyocytes (Fig. S2B). In contrast to the results from TNIP3 loss of function, both mRNA and protein expression levels of hypertrophic markers were remarkably downregulated by TNIP3 overexpression under PE conditions (Fig. S2C, D). Accordingly, in comparison with the adenoviral expressing green fluorescent protein (AdGFP) PE group, a reduced enrichment of cardiomyocyte hypertrophy and protein processing-related genes was evident in the GSEA analysis of the Ad*Tnip3* PE group within the RNA-sequencing profiles (Fig. S2E, F). Overall, TNIP3 alleviated PE-induced cardiomyocyte hypertrophy.

### TNIP3 interacts with STAT1 and promotes its total and phosphorylation levels during cardiac hypertrophy

**Fig. 6:**
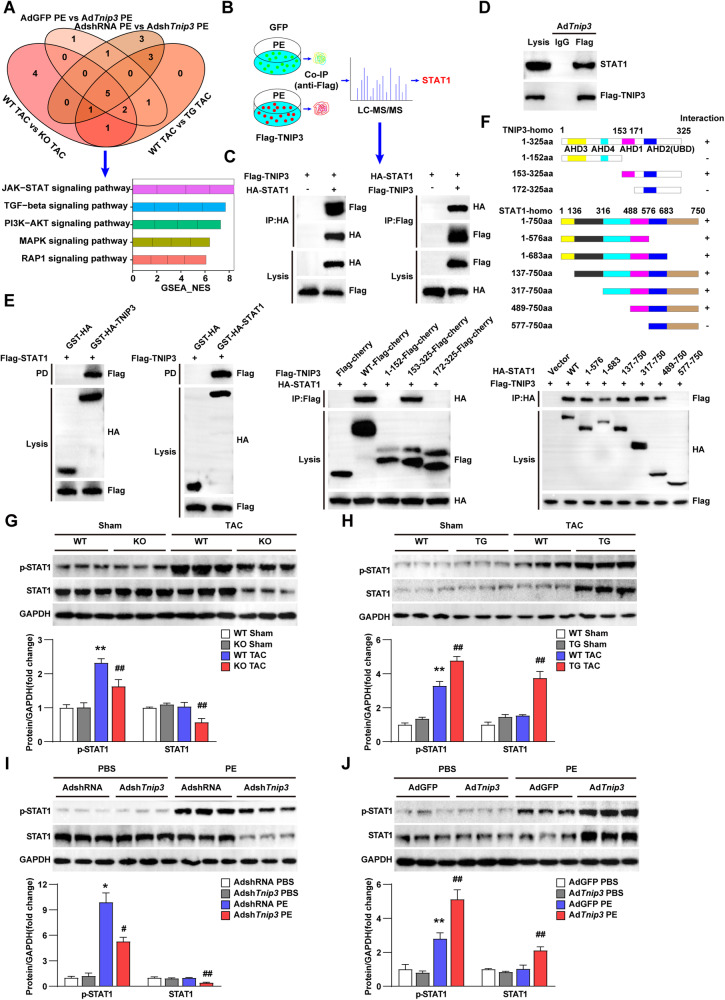
STAT1 serves as a potential downstream target of TNIP3. **A** Venn diagram of signaling pathway after combined analysis of four RNA sequencing experiments in vivo and in vitro and GSEA of five combined analysis signaling pathway based on KEGG database. **B** Schematic diagram showing MS analysis to identify the specific target of TNIP3. Co-immunoprecipitation assay of the interaction between TNIP3 and STAT1 in HEK293T cell transfected with the indicated plasmids (**C**) or cardiomyocytes infected with the indicated adenovirus (**D**). **E** GST pull-down assays showing the direct binding of TNIP3 and STAT1. Purified GST was used as a control. **F** Schematics of the human TNIP3 and STAT1 full-length and fragments constructs and interaction domain of human TNIP3 and STAT1 as well as their related truncated mutants. **G**–**J**, Representative western blots (upper) images of p-STAT1, STAT1 and quantification (bottom) from indicated groups (*n* = 3). *P < 0.05 or **P < 0.01 vs WT Sham group or AdshRNA PBS group or AdGFP PBS group, ^#^*P* < 0.05 or ^##^*P* < 0.01 vs WT TAC group or AdshRNA PE group or AdGFP PE group. Statistical analysis was carried out by one-way ANOVA.

To further reveal the mechanism of TNIP3 on cardiac hypertrophy, we combined analysis of RNA-sequencing in heart tissues and cardiomyocytes to search for potential targets of TNIP3 based on Kyoto Encyclopedia of Genes and Genomes (KEGG) database. The results showed that five signaling pathways were significantly activated, and Janus kinase /STAT(JAK/STAT) signaling pathway was the top pathway affected by TNIP3 (Fig. 6A). Meanwhile, we conducted co-immunoprecipitation (co-IP) followed by LC-MS/MS to search for the potential interacting protein with TNIP3 and found that STAT1, a critical transcription factor in JAK/STAT signaling pathway, could interact with TNIP3. (Fig. 6B). The interaction between TNIP3 and STAT1 was validated by co-IP assays (Fig. 6C, D). Glutathione S-transferase (GST) pull-down assay further confirmed that TNIP3 directly bounds to STAT1 (Fig. 6E). A molecular mapping assay indicated that the 153-171 amino acid (aa) AHD1 domain of TNIP3 was responsible for the interaction with the 488-576 amino acid (aa) LD domain on STAT1(Fig. 6F). Furthermore, p-STAT1 was increased in both mouse hearts following TAC surgery and in cardiomyocytes stimulated by PE at specific time points, with no change observed in STAT1 under hypertrophic stress compared with the basal state (Fig. S4A, B). Additionally, our findings also suggest that TNIP3 promotes the expression of total and phosphorylation of STAT1 in hypertrophic models. However, no significant changes were observed in the protein levels of p-STAT1 and total STAT1 in mouse hearts subjected to sham surgery or cardiomyocytes stimulated by PBS. Furthermore, under hypertrophic stress, there was a significant increase in the levels of p-STAT1 compared to control groups, while total STAT1 levels remained unchanged (Fig. 6G–J). Simultaneously, TNIP3 significantly enhanced the transcription factor activity of STAT1 and activated its target genes, as demonstrated by luciferase assay under hypertrophic stress conditions (Fig. S5A, B). These data indicated that TNIP3 could bind to STAT1 directly and increase protein level of total and phosphorylated STAT1 in cardiac hypertrophy along with promoting transcription factor activity of STAT1.

### TNIP3 facilitates STAT1 stability by suppressing STAT1 K48 ubiquitination

**Fig. 7:**
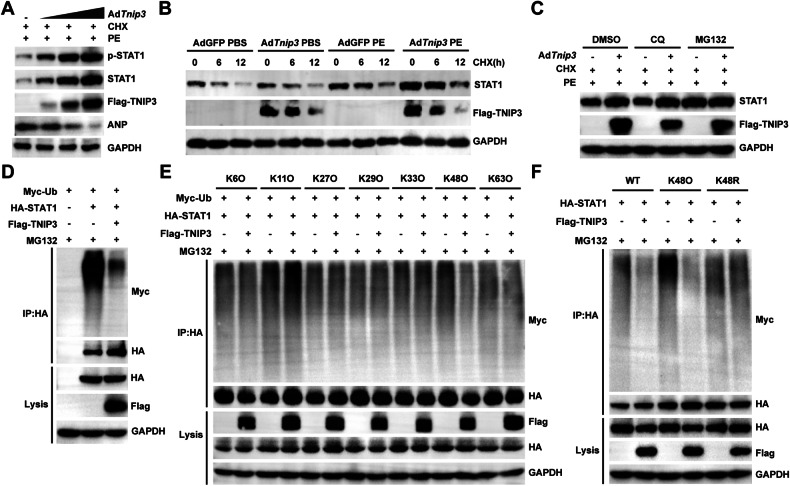
TNIP3 promotes STAT1 stability via inhibiting STAT1 K48 ubiquitination. **A** Representative western blots images of p-STAT1, STAT1, Flag-TNIP3 and ANP in NRCMs infected with the indicated adenovirus and incubated with PE (50 μM, 24 h) and CHX (50 μM, 6 h). **B** Immunoblotting analysis of STAT1 and Flag-TNIP3 from indicated groups. **C** Immunoblotting analysis of STAT1 and Flag-TNIP3 in indicated adenovirus infected-NRCMs treated with PE (50 μM, 24 h), DMSO, MG132 (50 μM) or CQ (50 μM) for 6 h. **D** Ubiquitination level of STAT1 in HEK293T cell co-transfected with indicated constructs for 24 h and incubated with MG132 (50 μM, 6 h). **E** Ubiquitination level of STAT1 with or without Flag-tagged *TNIP3* construct in HEK293T cell co-transfected with HA-tagged *STAT1* and indicated Myc-tagged ubiquitin (K6O, K11O, K27O, K29O, K33O, K48O, and K63O) constructs for 24 h and incubated with MG132 (50 μM, 6 h). **F** Ubiquitination level of STAT1 in MG132 (50 μM, 6 h) treated-HEK293T cell co-transfected with Flag-tagged *TNIP3*, HA-tagged *STAT1* and indicated Myc-tagged ubiquitin (WT, K48O, and K48R).

Considering the promotive effect of TNIP3 on total STAT1 protein during cardiac hypertrophy, we further investigated whether TNIP3 could affect the degradation of STAT1. We treated Ad*Tnip3-*infected cardiomyocytes with protein synthesis inhibitor cycloheximide (CHX) and the results indicated that TNIP3 overexpression inhibited endogenous STAT1 protein degradation and upregulated p-STAT1 during PE induced cardiomyocytes hypertrophy (Fig. 7A). In addition, we observed that overexpression of TNIP3 inhibited STAT1 degradation in a time-dependent manner under PE stimulation (Fig. 7B). However, this effect was reversed by the addition of MG132, a potent inhibitor of the proteasome (Fig. 7C). These results suggested that TNIP3 might boost STAT1 by inhibiting ubiquitin-proteasome system under pressure conditions. Subsequently, ubiquitination results validated that TNIP3 predominantly inhibited STAT1 K48-type ubiquitination (Fig. 7D-F). Taken together, TNIP3 suppressed STAT1 K48-type ubiquitination to stabilize STAT1.

### TNIP3 protects against cardiac hypertrophy via STAT1

**Fig. 8:**
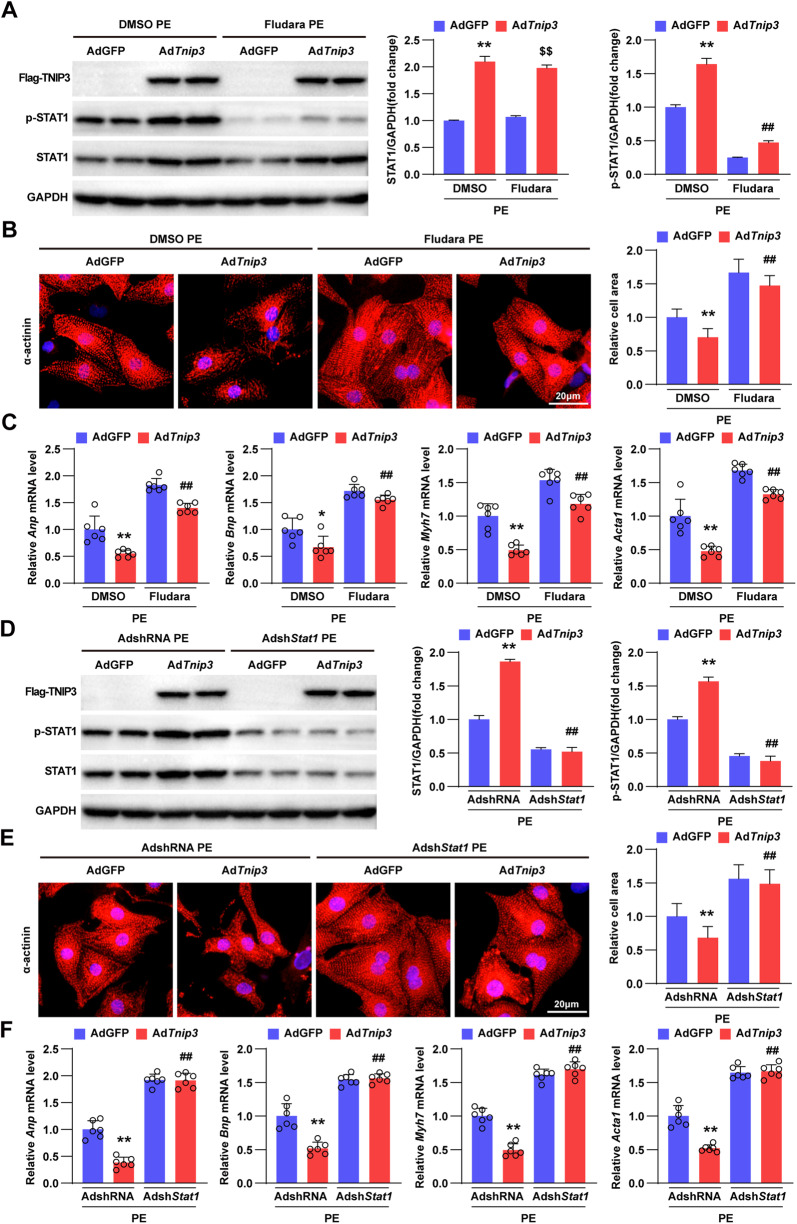
TNIP3 inhibits cardiac hypertrophy through STAT1. **A** Representative western blots (left) images of Flag-TNIP3, p-STAT1, STAT1 and quantification (right) in Ad*Tnip3* and AdGFP-infected NRCMs treated with or without Fludarabine (Fludara, STAT1 inhibitor, 5 μM, 24 h) under PE (50 μM, 24 h) incubation. **B** Representative images of α-actinin immunofluorescence staining (left) and quantification (right) from indicated groups (*n* > 50 cells). Scale bars, 20 μm. **C** Relative mRNA expression of hypertrophic marker genes from indicated groups (*n* = 6). **D** Representative western blots (left) images of TNIP3, p-STAT1, STAT1 and quantification (right) in Ad*Tnip3* and AdGFP-infected NRCMs treated with or without Adsh*Stat1* under PE (50 μM, 24 h) incubation. **E** Representative images of α-actinin immunofluorescence staining (left) and quantification (right) from indicated groups (*n* > 50 cells). Scale bars, 20 μm. **F** Relative mRNA expression of hypertrophic marker genes from indicated groups (*n* = 6). *P < 0.05 or **P < 0.01 vs AdGFP DMSO PE or AdGFP AdshRNA PE group, ^##^*P* < 0.01 vs Ad*Tnip3* DMSO PE or Ad*Tnip3* AdshRNA PE group, ^$$^*P* < 0.01 vs AdGFP Fludara PE. Statistical analysis was carried out by one-way ANOVA.

We further detected whether inactivation of STAT1 could block the regulation of TNIP3 on cardiomyocyte hypertrophy induced by PE. We treated Ad*Tnip3* infected NRCMs with inhibitor of STAT1, Fludarabine (Fludara) and the efficiency was confirmed by western blotting (Fig. 8A). Immunofluorescent staining for α‐actinin showed that inhibiting the activation of STAT1 remarkably promoted PE-induced cardiomyocyte hypertrophy as well as the expression of hypertrophic marker genes (Fig. 8B, C). Furthermore, we conducted adenoviral short hairpin RNA targeting *Stat1* (Adsh*Stat1*) to reduce STAT1 in cardiomyocytes infected by Ad*Tnip3*. The western blot results showed that the upregulation of total STAT1 and its activation in Ad*Tnip3*-infected cardiomyocytes were both blunted by Adsh*Stat1* under PE incubation (Fig. 8D). In accordance with western blots, diminution of cardiomyocytes stained by α‐actinin and downregulated expression of hypertrophic marker genes were also significantly blocked by STAT1 knockdown (Fig. 8E, F). Collectively, these data indicated that the reduction of STAT1 could abolish the protective effect of TNIP3 against cardiac hypertrophy.

## Discussion

Pathological cardiac hypertrophy is complex and related to increasing the risk of heart failure [16]. Although important molecular targets and signaling pathways involved in the development process of cardiac hypertrophy have been revealed in recent years [17–21], therapies in clinic targeted cardiac hypertrophy are still challenged. Our previous studies demonstrated that TNIP3 acts as a novel negative regulator of nonalcoholic steatohepatitis [14] and hepatic ischemia/reperfusion injury [15]. This study further investigated the function of TNIP3 in pathological cardiac hypertrophy. In this study, we found that the expression of TNIP3 was significantly increased in cardiac hypertrophy model in vivo and in vitro. Knockout of *Tnip3* in mice aggravated TAC induced cardiomyocyte hypertrophy, fibrosis, and cardiac function. Conversely, cardiac-specific overexpression of *Tnip3* markedly alleviated these phenotypic changes in the mouse heart. The regulatory effect of TNIP3 in hypertrophic NRCMs stimulated by PE presented same trends as in vivo.

The JAK/STAT signaling pathway has become recognized as a central mediator of widespread and various human physiological processes, including hematopoiesis, immune fitness, tissue repair, and adipogenesis [22]. STAT1 is a prominent transcription factor in JAK/STAT signaling pathway, which plays a role in numerous diseases, such as tumor [23–25], immune response activation [26], atherosclerosis [27], myocardial infarction [28], heart failure [29] and cardiac hypertrophy [30]. In canonical JAK/STAT1 signaling, binding of the ligands to the receptors induces receptor dimerization and activation of receptor associated JAK kinase, which in turn phosphorylates STAT1 or other STATs. Subsequently, STAT1 forms homo- or heterodimers and translocates to the nucleus to regulate gene expression [31]. To illustrate the mechanism of how TNIP3 regulates pathological cardiac hypertrophy, we combined RNA-Seq and mass spectrometry data to analyze and identify STAT1 to serve as the potential target of TNIP3. Importantly, our results also demonstrated that STAT1 interacts directly with TNIP3, and suppression of STAT1 activation or knockdown of STAT1 could block the protective effect of TNIP3 on PE-induced cardiomyocytes.

Our data also showed that TNIP3 increased protein level of STAT1 in cardiac hypertrophy and even in hypertrophic cardiomyocytes under cycloheximide treatment.

Therefore, we further investigated the role of TNIP3 in the degradation of STAT1 and found that TNIP3 affected STAT1 ubiquitination. Moreover, subsequent results indicated that TNIP3 repressed STAT1 K48-type ubiquitination to promote STAT1 stability. Previous studies revealed that TNIP3 could mediate multiple disease by targeting on deubiquitinase or ubiquitinase such as A20, TRIM8 and NEDD4 [13–15].

However, TNIP3 is an adapter protein without deubiquitinase activity in our study. Therefore how to clarify the exact mechanism of TNIP3 on ubiquitination of STAT1 is still needed for further investigation in the future.

In summary, this study demonstrates for the first time that TNIP3 plays an important role in protecting against pathological cardiac hypertrophy. Our results indicate that TNIP3 acts as a negative regulator by enhancing STAT1 stability through the inhibition of K48-type ubiquitination in response to hypertrophic stimulation. This finding suggests that TNIP3 may play a crucial role in modulating the cellular response to hypertrophic stimuli by regulating the stability of STAT1. Thus, targeting TNIP3-STAT1 might be a promising therapeutic target for pathological cardiac hypertrophy. These observations may provide new strategies and promising therapeutic target for pathological cardiac hypertrophy and heart failure via targeting of TNIP3.

### Supplementary information

Supplementary materials and figures

Original western blots

## Data Availability

All data in this paper are presented in the published article and its supplementary material files. Additional data related to this paper are available from the corresponding author on reasonable request.
