# Supplementary material for: TNIP3 protects against pathological cardiac hypertrophy by stabilizing STAT1
Source: Cell Death Dis. 2024 Jun 26;15(6):450. doi: 10.1038/s41419-024-06805-4 (PMC11208599; doi:10.1038/s41419-024-06805-4)
Supplement: Supplementary file 1 — Supplementary materials and figures [file 41419_2024_6805_MOESM1_ESM.docx]

TNIP3 Protects Against Pathological Cardiac Hypertrophy by Stabilizing STAT1

Hongjie Shi^1,2^*; Yongjie Yu^1,2^*; Dajun Li^1,2^*; Kun Zhu^1,2^; Xu Cheng^3,4^; Tengfei Ma^5,7^; Zhangqian Tao^2^; Ying Hong^2^; Zhen Liu^8^; Siyi Zhou ^1,2^; Jianqing Zhang^8^; Yun Chen^6^; Xiao-Jing Zhang^1,2^; Peng Zhang^1,2#^; Hongliang Li^1,2,3,4#^.

^1^ Taikang Medical School (School of Basic Medical Sciences), Wuhan University, 430000Wuhan, China.

^2^ Institute of Model Animal, Wuhan University, 430000Wuhan, China.

^3^ Gannan Innovation and Translational Medicine Research Institute, Gannan Medical University, 341000Ganzhou, China.

^4^ State Key Laboratory of New Drug Discovery and Development for Major Diseases, Gannan Innovation and Translational Medicine Research Institute, 341000Ganzhou, China.

^5^ Department of Neurosurgery, Huanggang Central Hospital, 438000Huanggang. China.

^6^ Clinical trial centers, Huanggang Central Hospital, 438000Huanggang. China.

^7^ Huanggang Institute of Translational Medicine, 438000Huanggang, China.

^8^ Department of Cardiology, Renmin Hospital of Wuhan University, 430000Wuhan, China.

*These authors contributed equally to this work.

# Correspondence authors.

Peng Zhang E-mail: zhp@whu.edu.cn

Hongliang Li E-mail: [lihl](mailto:dev@null)@whu.edu.cn

Address: Taikang Medical School (School of Basic Medical Sciences), Wuhan University, 115 Donghu Road, Wuchang District, 430000Wuhan, Hubei Province, China.

**MATERIALS AND METHODS**

**Animal models and procedures**

Global *Tnip3* knockout (KO) mice were generated following the methodology described in our previous work [11]. For the creation of cardiac-specific *Tnip3* transgenic (TG) mice, the full length of mouse *Tnip3* cDNA was cloned downstream of the cardiomyocyte-specific a-myosin heavy chain (a-MHC) promoter into the plasmid. The a-MHC-*Tnip3* plasmid was then linearized and microinjected into fertilized mouse embryos to obtain cardiomyocyte-specific *Tnip3* TG mice on a C57BL/6J background. Subsequently, mouse toe DNA was analyzed by polymerase chain reaction (PCR) using the following primers to confirm the presence of the *Tnip3* transgene: forward: 5’-CATAGAAGCCTAGCCCACACCA-3’, and reverse: 5’-GGACAAGGCTGGTGGGC A-3’. All mice used in the experiments were housed in a specific pathogen-free environment with stable temperature, humidity, and light cycles (12 hours light/12 hours dark), and had ad libitum access to food and water. *Tnip3*-KO mice and their wild-type (WT) littermates, as well as *Tnip3* TG and WT littermate male mice, were utilized in subsequent experiments.

Transverse aortic constriction (TAC) surgery was performed to establish a mouse model of pressure overload-induced pathological cardiac hypertrophy, as previously described [14,16]. Briefly, male mice aged 9-11 weeks and weighing 25.5-27g were randomly selected and anesthetized by Zoletil®50 (VIRBAC, France) at a dosage of 12.5 mg/kg per mouse until the disappearance of toe contractile reflex on an auto-adjusting heating pad maintaining body temperature at 37℃. Following the incision of the mice's skin and muscles at the midpoint between the clavicle and thoracic vertebrae, the aortic arch was exposed beneath the thymus. TAC surgery was conducted by ligating the transverse aorta with a 26-gauge needle using 7-0 silk sutures. Subsequently, the needle was removed before suturing the skin. The sham group underwent identical procedures without aortic ligation. All surgeries were performed under blinding. There was no statistical procedure employed to calculate the sample size for the mouse experiment, which was relied on preliminary experimental data. The sample size for each experiment is provided in the legends.

**Echocardiographic measurements**

Echocardiographic analyses were blindly performed to evaluate cardiac function at the specified time points, following previously described methods [9]. Briefly, the mice were anesthetized with 1.5-2% isoflurane, and the Small Animal Ultrasound Imaging System (VEVO2100, FUJIFILM VISUALSONICS) equipped with a 30-MHz (MS400) probe was utilized to acquire at least three consecutive cardiac cycles of left ventricular volume and left ventricular wall thickness using M-mode tracings at the level of the papillary muscles. The echocardiographic parameters assessed included heart rate (HR), left ventricular end-systolic diameter (LVESd), left ventricular end-diastolic diameter (LVEDd), left ventricular ejection fraction (LVEF), and left ventricular fractional shortening (LVFS). These parameters were calculated to provide comprehensive insights into the cardiac function.

**Histological analyses**

After 4 weeks of sham or TAC surgery, mice were euthanized upon cessation of respiration. The body weight of each mouse was recorded, and the hearts, lungs, and tibiae were collected for further analysis. The hearts were rinsed in 10% KCL solution, then fixed in 10% formalin, and embedded in paraffin using standard histological protocols. Subsequently, the hearts were transversely sectioned at a thickness of 5μm and stained with hematoxylin-eosin (H&E) to assess myocyte hypertrophy and picrosirius red (PSR) to evaluate cardiac fibrosis. Additionally, Alexa Fluor® 488 wheat germ agglutinin (WGA) staining was performed to detect myocyte cross-sectional areas (CSA), while nuclei were labeled with DAPI. Imaging was conducted using Digital Pathology Slide Scanners (Aperio Versa 200, Leica) and fluorescence microscope (BX51, Olympus). The CSA and left ventricular (LV) collagen volume were quantified through a digital image analysis system (Image-Pro Plus Version 6.0), with measurements of at least 100 cardiomyocytes and more than 40 fields in each group for CSA and LV collagen volume quantification, respectively.

**Immunohistochemistry (IHC)**

The mouse heart tissue sections were subjected to antigen retrieval and subsequently washed in 3% H_2_O_2_ at room temperature for 20 minutes. Following this, the sections were blocked with 10% bovine serum albumin (BSA) at 37°C for 30 minutes. The TNIP3 primary antibody (BT-AP15046, 1:100 dilution, BT LAB) was then applied and the sections were incubated at 4°C overnight. Subsequently, the sections were washed with PBS and treated with the Goat anti-Rabbit Detection Kit (BLRE006-200T, Biolight) at 37°C for 1 hour. Positive signals on the slices were visualized using diaminobenzidine working solution (ZLI-9018, ZSGB-BIO) after washing with PBS. Hematoxylin staining (G1004, Servicebio) was performed, followed by washing with ddH_2_O. Finally, the slices were sealed with a resin sealant (BA-7004, Baso), and images were obtained using Digital Pathology Slide Scanners (Aperio Versa 200, Leica).

**Primary neonatal rat cardiomyocytes (NRCMs) culture and adenovirus infection**

NRCMs were isolated from 1-2-day-old Sprague-Dawley rats following the method described [14]. Briefly, the heart tissue was dissected into 1-2 mm³ pieces and digested with 0.125% trypsin to facilitate cell isolation. Following separation using the differential attachment technique, the isolated cardiomyocytes were cultured in DMEM/F-12 (C11330500BT, Gibco) medium supplemented with 10% fetal bovine serum (FBS) (10099141C, Gibco), 1% penicillin/streptomycin (15140-122, Gibco), and 0.1mM 5-Bromo-2′-deoxyuridine (BrdU) (B5002-250MG, Sigma) for an overnight incubation period of 24 hours. Subsequently, the cardiomyocytes were transitioned to a serum-free culture medium for 12 hours prior to stimulation with 50μM phenylephrine (PE) (PHR1017, Sigma) or an equivalent volume of phosphate-buffered saline (PBS) for the specified time points. In the case of cardiomyocytes treated with a STAT1 inhibitor, 5μM Fludarabine (Fludara) (MedChemExpress) was co-administered with PE stimulation. NRCMs were infected with corresponding adenoviruses at a multiplicity of infection (MOI) of 50 particles per cell for 6 hours in subsequent experiments.

**Immunofluorescence staining**

NRCMs were stained with α-actinin antibody（05-384, Merck Millipore, 1:100 dilution）to estimate size of cardiomyocytes. Briefly, NRCMs were fixed in 4% formaldehyde (G1101-500ML, Servicebio) for 30 min after PE stimulation for 24 hours. After washing in PBS, the cardiomyocytes were permeabilized with 0.2% Triton X-100 (T8787, Sigma-Aldrich) for 5 min and blocked with 8% goat serum (BMS0050, Abbkine) at 37℃. Then cardiomyocytes on slide were incubated with α-actinin antibody, then treated with secondary antibody (A21202, Invitrogen, 1:200 dilution) subsequently. Finally, images were captured under fluorescence microscope (BX51, Olympus).

**Quantitative real-time PCR (qPCR)**

Total RNA was extracted from mouse heart tissues and cultured NRCMs by TRIzol reagent (15596-026, Invitrogen). HiScript III RT SuperMix for qPCR (R323-01, Vazyme) was applied to convert mRNA to cDNA. Then mRNA expression of target genes was detected by ChamQ SYBR qPCR Master Mix (Q311-02, Vazyme) and LightCycler480 II Instrument (Roche) under a standard procedure. Glyceraldehyde-3-phosphate dehydrogenase (*Gapdh*) was used as the reference gene and the primer sequence of target genes are shown in Supplementary Table 1.

**Western blot analysis**

For western blot analysis, samples from mouse hearts and cardiomyocytes were split in

RIPA lysis buffer (65 mM Tris-HCl pH 7.5, 150 mM NaCl, 1 mM EDTA, 1 % NP-40 (N8030, Solarbio), 0.5% sodium deoxycholate, 0.1% SDS) containing protease inhibitor to obtain total protein. BCA protein assay kit (23225, Thermo) was applied to determine protein concentration. After separating the quantities of equal extracted protein by SDS-PAGE, the separated protein was transferred to PVDF membranes (IPVH00010, Millipore) from gel and blocked with 5% skim milk in TBST (Tris-buffered saline and 0.1% Tween-20) for 1hour. Then PVDF membranes were incubated with indicated primary antibodies overnight at 4°C followed by appropriate secondary antibodies for 1 hour at room temperature. Eventually the target protein in PVDF membranes were incubated with enhanced chemiluminescence (ECL) reagents (170-5061, Bio-Rad) and detected by ChemiDoc MP Imaging System (Bio-Rad). Quantification of protein was conducted by Image J software. GAPDH was served as a loading control and all used antibodies are shown in Supplementary Table 2.

**Plasmids and recombinant adenoviruses construction**

Plasmids encoding full-length region of human *TNIP3* and truncated *TNIP3* were constructed as previously described [10]. Full-length region of human *STAT1* and truncated *STAT1* were cloned into PcDNA5-Flag vector. For adenoviral overexpression TNIP3(Ad*Tnip3*), rat full-length *Tnip3* were cloned in replication deficient adenoviral vectors with CMV promoter. We generated rat short hairpin RNA against *Tnip3 or Stat1* adenoviral (Adsh*Tnip3 or* Adsh*Stat1*)*.* Similar adenoviral expression green fluorescent protein (AdGFP) and non-targeting short hairpin RNA (AdshRNA) regarded as controls. Primers used for plasmids and recombinant adenoviruses construction are listed in Supplementary Table 3.

**Luciferase Reporter Assays**

The luciferase reporter assay was conducted in NRCMs as previously described. An adenovirus encoding a STAT1-dependent luciferase reporter (Ad*Stat1*-luc) was constructed, which contains three copies of a STAT1 binding site followed by the firefly luciferase reporter gene. Then the cardiomyocytes were infected with Ad*Stat1*-luc as well as Adsh*Tnip3* or Ad*Tnip3* for 6 hours. After infection, the cardiomyocytes were incubated with either PE or PBS for 24 hours before being harvested. The cardiomyocytes were washed three times and then lysed in passive lysis buffer (Promega). The luciferase activity in the cells was then determined using the Dual-Luciferase Reporter Assay System (Promega).

**Immunoprecipitation (IP) assay**

After indicated plasmids co-transfected HEK293T cells (GDC0187, China Center For Type Culture Collection) and corresponding adenoviruses infected NRCMs, cells were treated with cold IP lysis buffer (20 mM Tris HCl, pH 7.4; 150 mM NaCl; 1 mM EDTA; and 1%Triton X-100) containing protease inhibitor (04693132001, Roche) and phosphatase inhibitor (4906837001, Roche). Samples were centrifuged at 4°C for 10 min and the supernatant containing protein was incubated with protein G Bestarose 4FF beads (AA104307, Bestchrom) and indicated labeled antibody overnight at 4°C. Finally, the beads were washed with cold IP buffer containing 150mM or 300mM NaCl for three times respectively. Then the beads were boiled in 2x SDS loading buffer at 95°C for 10min prior to western blotting analysis. To conduct IP-mass spectrometry analysis, the precipitated proteins were separated by SDS-PAGE gels and then delivered to Shang Hai Bioprofile Technology for detection.

**Glutathione S-transferase (GST) pull-down assay**

Lysis of HEK293T cells transfected with indicated plasmids was obtained with lysis buffer (50mM Na_2_HPO4, 300mM NaCl, 1% Triton) containing protease inhibitor cocktail tablets (04693132001, Roche) and purified by Glutathione Sepharose 4B beads (45-000-139, GE Healthcare), Flag antibodies and Flag peptide (Sigma, F4799). Purified GST-HA tagged protein was incubated with Glutathione Sepharose 4B beads for 3 hours at 4°C before mixed with purified Flag tagged protein for overnight at 4°C. The beads were washed by cold GST buffer (20mM Tris-HCl pH 6.8, 150mM NaCl, 0.2% TritonX-100) for three times and western blotting analysis was conducted after precipitated proteins boiled in 2x SDS loading buffer at 95°C for 10min.

**Ubiquitination assay**

HEK293T cells transfected with indicated plasmids were lysed with SDS lysis buffer (20 mM Tris–HCl, pH 7.4, 150 mM NaCl,1 mM EDTA, 1% SDS) containing protease inhibitor cocktail and boiled at 95°C for 10min. Then IP buffer (20 mM Tris HCl, pH 7.4; 150 mM NaCl; 1 mM EDTA; and 1%Triton X-100) was added to dilute lysates. After centrifuged at12,000 rpm for 10 min at 4°C, the supernatants were detected by IP assay with indicated antibodies.

**RNA-Sequencing and data processing**

RNA extracted from mouse heart tissues subjected to TAC surgery and cultured NRCMs stimulated with PE was obtained to constructed cDNA library by MGI Easy RNA Library Prep Kit (1000006384, MGI). BGISEQ-2000RS (MGI) instrument was performed to analyze gene expression profiling and HISAT2 software was conducted mapped sequencing fragments to mouse (mm10/GRCm38) or rat (rn6/Rnor_6.0) reference genome after data cleaning. Binary Alignment Map (BAM) files were acquired by SAM tools software. Then fragments per kilobase of exon model per million mapped fragments (FPKM) of each identified gene was calculated by String Tie software. Subsequently differentially expressed genes (DEGs) were screened by DESeq2 software depending on two factors: fold change＞2 and the relevant adjusted *P* values＜0.05. The R package gmodels was used for principal component analysis (PCA).

**Kyoto Encyclopedia of Genes and Genomes (KEGG) enrichment analysis**

All DGEs were analyzed for enrichment in signaling pathways using statistical methods such as Fisher's exact test and were annotated into pathways available in the KEGG database. Signaling pathways with a *P* value < 0.05 were considered statistically enriched.

**Gene set enrichment analysis (GSEA)**

For each Gene Ontology (GO) biological process term, the genes involved were defined as a gene set. The gene sets were then analyzed using GSEA on the Java GSEA platform. Statistical significance was determined based on a *P* value < 0.05 and a false discovery rate (FDR) value < 0.25.

**Statistical analysis**

The data in this study were analyzed using Statistical Package for the Social Sciences 25.0 (SPSS 25.0) and presented as mean ± standard deviation (SD). Data visualization was performed using GraphPad Prism 8.0, and normal distribution of the data was assessed using the Shapiro-Wilk test. Two-tailed Student’s t-test was utilized for comparison between two groups, while one-way ANOVA was conducted to compare for more than two groups with Bonferroni post hoc analysis (data meeting homogeneity of variance) or with Tamhane T2 analysis (data of heteroscedasticity) when data conformed to a normal distribution. A *P* value < 0.05 was considered statistically significant.

**DATA AVAILABILITY**

All data in this paper are presented in the published article and its supplementary material files. Additional data related to this paper are available from the corresponding author on reasonable request.

**FigureS1**


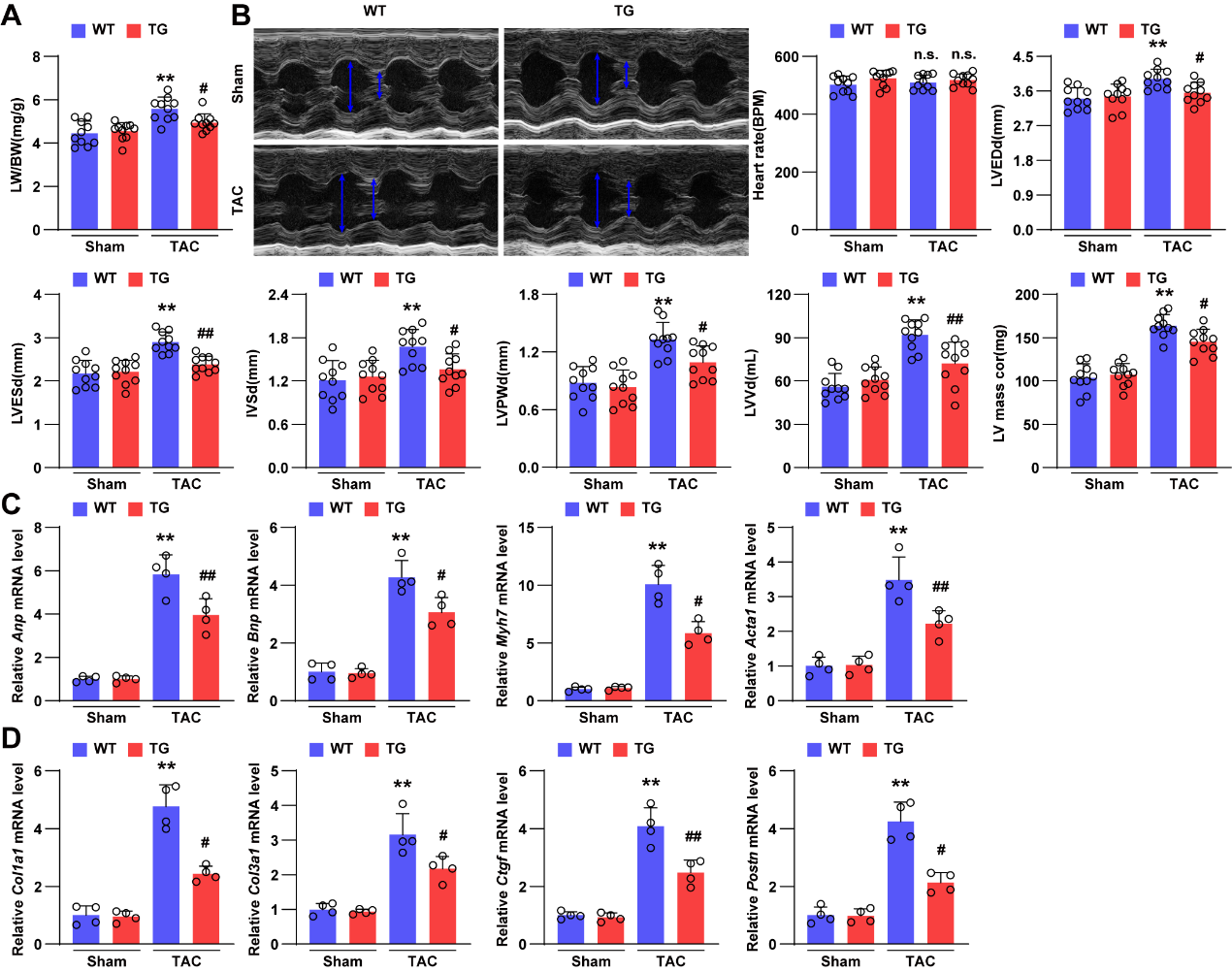


**FigureS1. Cardiac-specific overexpression of TNIP3 attenuates pathological cardiac hypertrophy.**

A, LW/BW in WT and TG mice at 4 weeks post sham or TAC surgery (n=10). B, Echocardiography images and parameters for HR, LVEDd, LVESd, IVSd, LVPWd, LVVd, and LV mass cor in each group (n=10). C-D, The relative mRNA expressions of hypertrophic marker genes (C) and fibrosis marker genes (D) in heart tissues in each group (n=4).  ^n.s.^*P*≥0.05 or ***P*<0.01 vs WT Sham group, ^n.s.^*P*≥0.05 or ^#^*P*<0.05 or ^##^*P*<0.01 vs WT TAC group. Statistical analysis was carried out by one-way ANOVA.

**FigureS2**


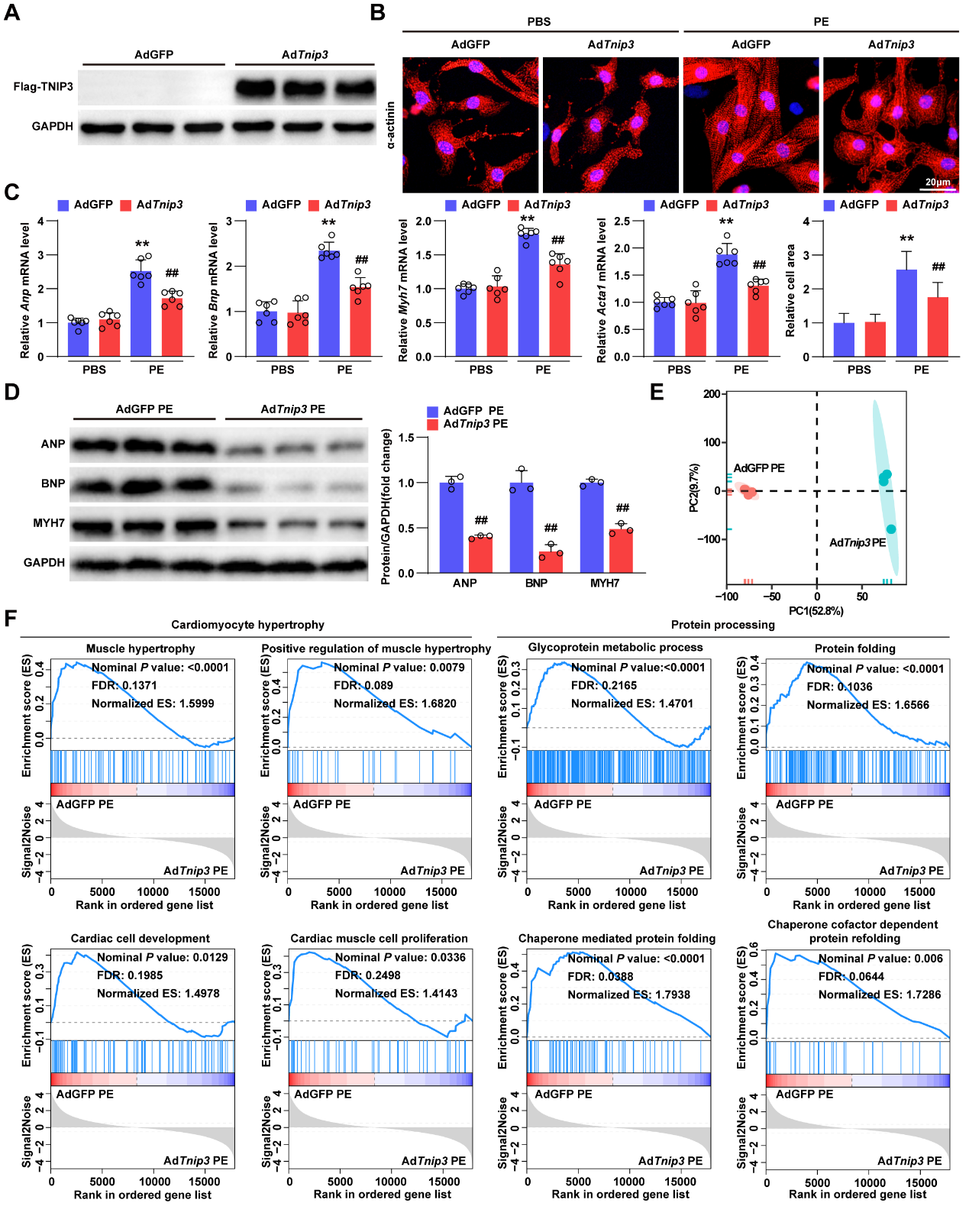


**FigureS2. TNIP3 overexpression relieves phenylephrine-induced cardiomyocyte hypertrophy.**

A, Representative Western blots of TNIP3 in NRCMs infected by Ad*Tnip3* (n=3). B, Representative images of α-actinin immunofluorescence staining (upper) and quantification (bottom) in Ad*Tnip3* and AdGFP-infected NRCMs treated with PBS or PE (50 μM) for 24h (n > 50 cells). Scale bars, 20μm. C, Relative mRNA expressions of hypertrophic marker genes in each group (n=6). D, Representative western blots (left) images and quantification (right) of hypertrophic marker from indicated groups. E, PCA of sample distribution profiles from indicated groups based on the RNA-seq (n=4). F, GSEA showed the majority enriched genes involved in cardiomyocyte hypertrophy and protein processing from indicated groups. ***P*<0.01 vs AdGFP PBS group, ^##^*P*<0.01 vs AdGFP PE group. Statistical analysis was carried out by one-way ANOVA and Two-tailed Student’s t-test.

**FigureS3**


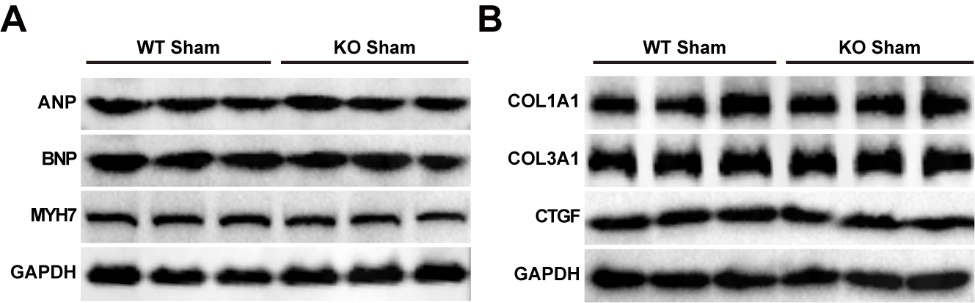


**FigureS3. TNIP3 deletion makes no difference on the expression of hypertrophic markers and fibrosis marker molecules under normal condition.**

A, Representative western blots of hypertrophic markers in sham groups (n=3). B, Representative western blots of fibrosis marker in sham groups (n=3).

**FigureS4**


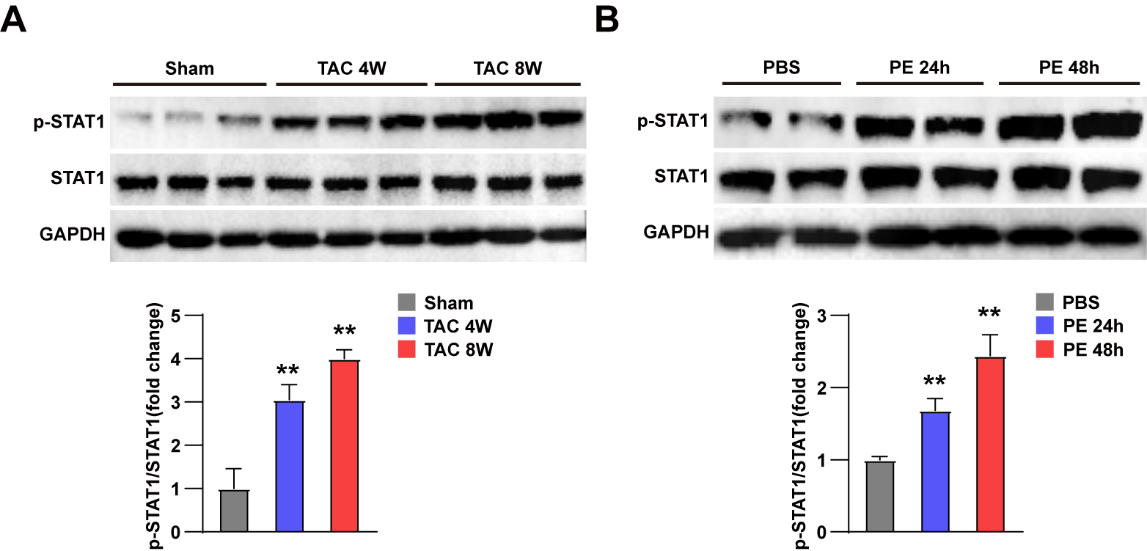


**FigureS4. STAT1 is activated in cardiac hypertrophy model.**

A, Representative western blots (upper) and quantification (bottom) of p-STAT1 and STAT1 expression in heart tissues in each group (n=3). B, Representative western blots (upper) and quantification (bottom) of p-STAT1 and STAT1 expression from indicated groups. ***P*<0.01 vs Sham or PBS group. Statistical analysis was carried out by one-way ANOVA.

**FigureS5**


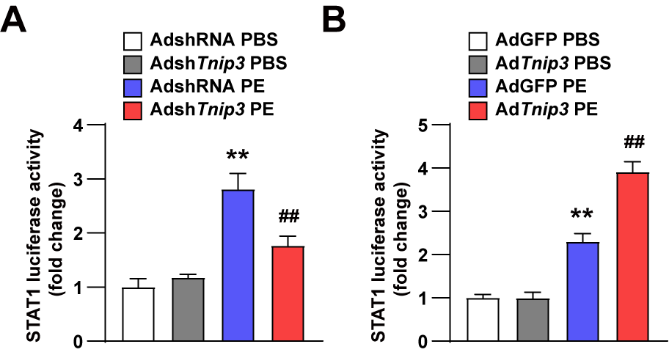


**FigureS5. TNIP3 promotes transcription factor activity of STAT1 under phenylephrine stimulation.**

A, STAT1‐luciferase activity in Adsh*Tnip3* and AdshRNA-infected NRCMs treated with or without PE (50 μM, 24h) incubation. B, STAT1‐luciferase activity in Ad*Tnip3* and AdGFP-infected NRCMs treated with or without PE (50 μM, 24h) incubation. ***P*<0.01 vs AdshRNA PBS group or AdGFP PBS group, ^##^*P*<0.01 vs AdshRNA PE group or AdGFP PE group. Statistical analysis was carried out by one-way ANOVA.

Supplementary table1

Primer sequences for RT-PCR detection

| Gene name | Forward primer | Reverse primer |
| --- | --- | --- |
| *Anp-*mouse | TCGGAGCCTACGAAGATCCA | TTCGGTACCGGAAGCTGTTG |
| *Bnp-*mouse | GAAGGACCAAGGCCTCACAA | TTCAGTGCGTTACAGCCCAA |
| *Myh7-*mouse | CAACCTGTCCAAGTTCCGCA | TACTCCTCATTCAGGCCCTTG |
| *ACTA1-*mouse | CCCACAACGTGCCCATCTAT | TCTCACGTTCAGCTGTGGTC |
| *Col1α1-*mouse | TGCTAACGTGGTTCGTGACCGT | ACATCTTGAGGTCGCGGCATGT |
| *Col3α1-*mouse | ACGTAAGCACTGGTGGACAG | CCGGCTGGAAAGAAGTCTGA |
| *Ctgf-*mouse | TGACCCCTGCGACCCACA | TACACCGACCCACCGAAGACACAG |
| *Postn-*mouse | GCAAACCACTTTCACCGACC | CGTTGGTCCATGCTCAGAGT |
| *Gapdh-*mouse | ACTCCACTCACGGCAAATTC | TCTCCATGGTGGTGAAGACA |
| *Anp*-rat | AAAGCAAACTGAGGGCTCTGCTCG | TTCGGTACCGGAAGCTGTTGCA |
| *Bnp*-rat | TGCCCCAGATGATTCTGCTC | TGTAGGGCCTTGGTCCTTTG |
| *Myh7*-rat | AGTTCGGGCGAGTCAAAGATG | CAGGTTGTCTTGTTCCGCCT |
| *ACTA1*-rat | TATCGGTATGGAGTCTGCGG | GATCCACACTGAGTACTTGCG |
| *Gapdh*-rat | CAGTGCCAGCCTCGTCTCAT | AGGGGCATCCACAGTCTTC |

Supplementary table2

Antibody for western blotting

| Anbibody | Manufacturer | Catalogue number | Source of species | Dilution |  |
| --- | --- | --- | --- | --- | --- |
| TNIP3 | BT LAB | BT-AP15046 | rabbit | 1:1000 |  |
| STAT1 | CST | 9172 | rabbit | 1:1000 |  |
| p-STAT1 | ABclonal | AP0054 | rabbit | 1:1000 |  |
| ANP | Proteintech | 27426-1-AP | rabbit | 1:1000 |  |
| BNP | ABclonal | A2179 | rabbit | 1:1000 |  |
| MYH7 | Proteintech | 22280-1-AP | rabbit | 1:1000 |  |
| COL1A1 | ABclonal | A16891 | rabbit | 1:1000 |  |
| COL3A1 | santa cruz | sc-271249 | mouse | 1:200 |  |
| CTGF | santa cruz | sc-14939 | goat | 1:200 |  |
| Flag | MBL | M185-3LL | rabbit | 1:1000 |  |
| HA | MBL | M180-3 | mouse | 1:1000 |  |
| Myc | MBL | M047-3 | mouse | 1:1000 |  |
| GAPDH | CST | 2118 | mouse | 1:5000 |  |

Supplementary table3

Primer sequence for construction of adenovirals and plasmids

| Gene name |  | | Reverse primer |
| --- | --- | --- | --- |
| Ad*Tnip3* | Forward primer: | GCTAGCGATATCGGATCCATGGCTGGACTGAGTGGTGC | |
|  | Reverse primer: | ACTAGTGGTACCAAGCTTCAGGTTGAATTTGCTTTCTGAGG | |
| Adsh*Tnip3* | Forward primer: | CCGGATGGGACCAGCAATTTCGAAACTCGAGTTTCGAA  ATTGCTGGTCCCATTTTTTG | |
|  | Reverse primer: | AATTCAAAAAATGGGACCAGCAATTTCGAAACTCGAG  TTTCGAAATTGCTGGTCCCA | |
| Adsh*Stat1* | Reverse primer: | CCGGGGTCTTATTCCATGGACAAGGCTCGAGCCTTGTC  CATGGAATAAGACCTTTTTG | |
|  | Reverse primer: | AATTCAAAAAGGTCTTATTCCATGGACAAGGCTCGAG  CCTTGTCCATGGAATAAGAACC | |
| *Flag-TNIP3* | Forward primer: | CGCGGATCCATGGCACATTTTGTACAGGG | |
|  | Reverse primer: | CCGCTCGAGCTACGGATGGACTTTCTTTACTGAG | |
| *GST-HA- TNIP3* | Forward primer: | CGCGGATCCATGGCACATTTTGTACAGGG | |
|  | Reverse primer: | CCGCTCGAGCTACGGATGGACTTTCTTTACTGAG | |
| *Flag- TNIP3 (1-152)* | Forward primer: | CGCGGATCCATGGCACATTTTGTACAGGG | |
|  | Reverse primer: | CCGCTCGAGCTACTTTTCCTTGTTCGCAAGAGTAT | |
| *Flag- TNIP3 (153-325)* | Forward primer: | CGCGGATCCGAACATTACGAATGTGAAATAA | |
|  | Reverse primer: | CCGCTCGAGCTACGGATGGACTTTCTTTACTGAG | |
| *Flag- TNIP3 (172-325)* | Forward primer: | CGCGGATCCATCAAGTGTTCATTTTCC | |
|  | Reverse primer: | CCGCTCGAGCTACGGATGGACTTTCTTTACTGAG | |
| *HA-STAT1* | Forward primer: | TCGGGTTTAAACGGATCCATGTCTCAGTGGTACG  AACTTCAGC | |
|  | Reverse primer: | GGGCCCTCTAGACTCGAGCTATACTGTGTTCATCAT  ACTGTCGAATTCTACAG | |
| *Flag-STAT1* | Forward primer: | TCGGGTTTAAACGGATCCATGTCTCAGTGGTACGAA  CTTCAGC | |
|  | Reverse primer: | GGGCCCTCTAGACTCGAGCTATACTGTGTTCATCATA  CTGTCGAATTCTACAG | |
| *GST-HA- STAT1* | Forward primer: | TCGGGTTTAAACGGATCCATGTCTCAGTGGTACGAAC  TTCAGC | |
|  | Reverse primer: | GGGCCCTCTAGACTCGAGCTATACTGTGTTCATCATAC  TGTCGAATTCTACAG | |
| *Flag-STAT1(137-750)* | Forward primer: | TCGGGTTTAAACGGATCCATGGACAAACAGAAAGAGC  TTGACAGTAAAGT | |
|  | Reverse primer: | GGGCCCTCTAGACTCGAGTACTGTGTTCATCATACTGT  CGAATTCTACAGAG | |
| *Flag-STAT1(317-750)* | Forward primer: | TCGGGTTTAAACGGATCCATGTTTGTGGTGGAAAGAC  AGCCCT | |
|  | Reverse primer: | GGGCCCTCTAGACTCGAGTACTGTGTTCATCATACTGT  CGAATTCTACAGAG | |
| *Flag-STAT1(489-750)* | Forward primer: | TCGGGTTTAAACGGATCCATGACTCCACCATGTGCAC  GATG | |
|  | Reverse primer: | GGGCCCTCTAGACTCGAGTACTGTGTTCATCATACTGT  CGAATTCTACAGAG | |
| *Flag-STAT1(577-750)* | Forward primer: | TCGGGTTTAAACGGATCCATGTGCATCATGGGCTTCAT  CAGCA | |
|  | Reverse primer: | GGGCCCTCTAGACTCGAGTACTGTGTTCATCATACTGT  CGAATTCTACAGAG | |
| *Flag-STAT1(1-576)* | Forward primer: | TCGGGTTTAAACGGATCCATGTCTCAGTGGTACGAAC  TTCAGC | |
|  | Reverse primer: | GGGCCCTCTAGACTCGAGCCCATCATTCCAGAGAGGG  AGC | |
| *Flag-STAT1(1-683)* | Forward primer: | TCGGGTTTAAACGGATCCATGTCTCAGTGGTACGAAC  TTCAGC | |
|  | Reverse primer: | GGGCCCTCTAGACTCGAGCCTGGAGTAATACTTTCCA  AAGGCAT | |
