# Supplementary material for: TNIP3 protects against pathological cardiac hypertrophy by stabilizing STAT1
Source: Cell Death Dis. 2024 Jun 26;15(6):450. doi: 10.1038/s41419-024-06805-4 (PMC11208599; doi:10.1038/s41419-024-06805-4)

Figure1

C

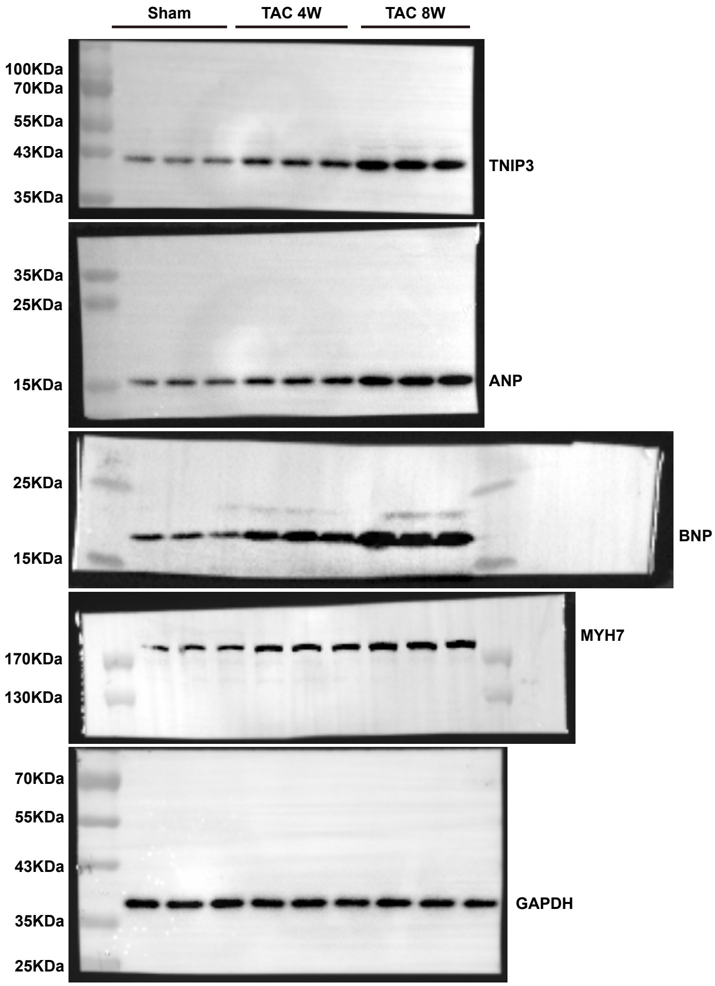

Sham
TAC 4W
TAC 8W
100KDa
70KDa
55KDa
43KDa
35KDa
TNIP3
35KDa
25KDa
15KDa
ANP
25KDa
15KDa
BNP
MYH7
170KDa
130KDa
70KDa
55KDa
43KDa
35KDa
25KDa
GAPDH

G

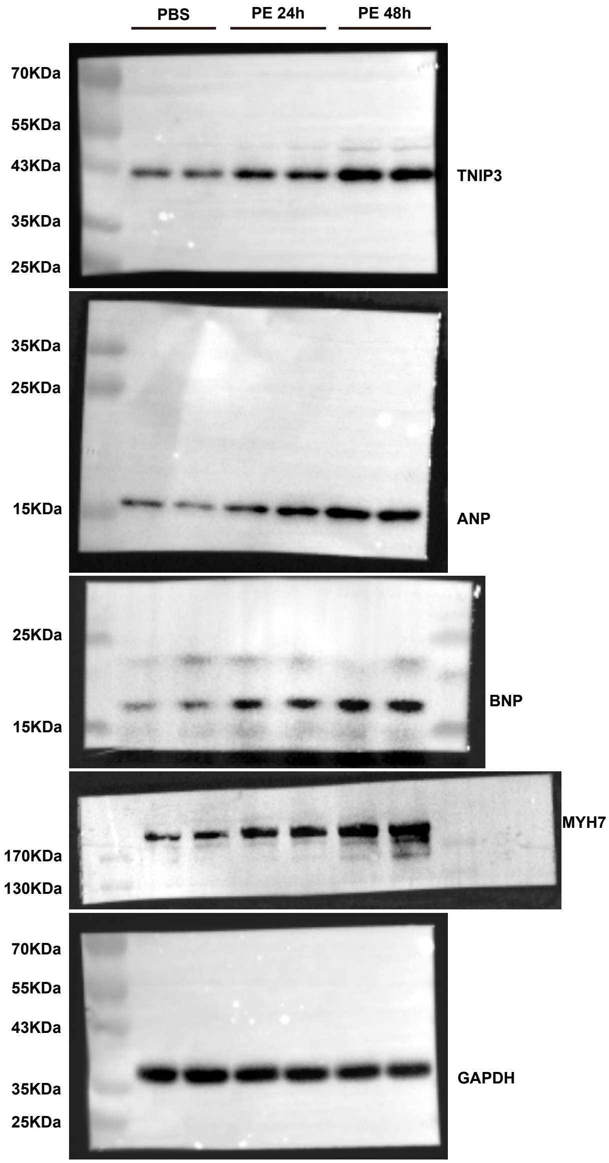

PBS
PE 24h
PE 48h
70KDa
55KDa
43KDa
35KDa
25KDa
TNIP3
35KDa
25KDa
15KDa
ANP
25KDa
15KDa
BNP
MYH7
170KDa
130KDa
70KDa
55KDa
43KDa
35KDa
25KDa
GAPDH

Figure2

A

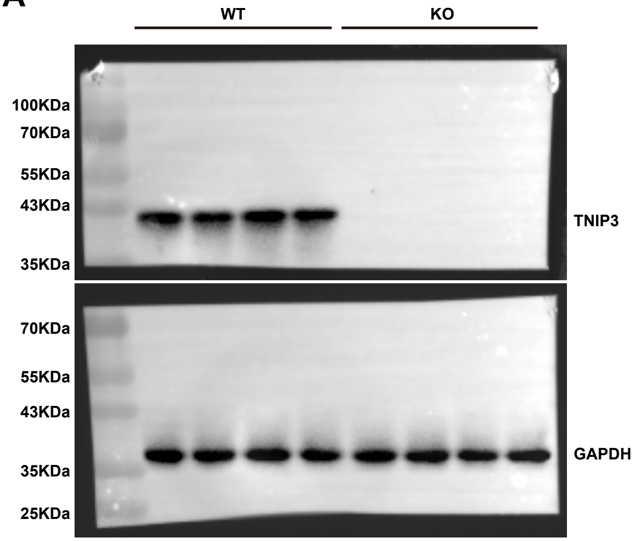

Figure3

C

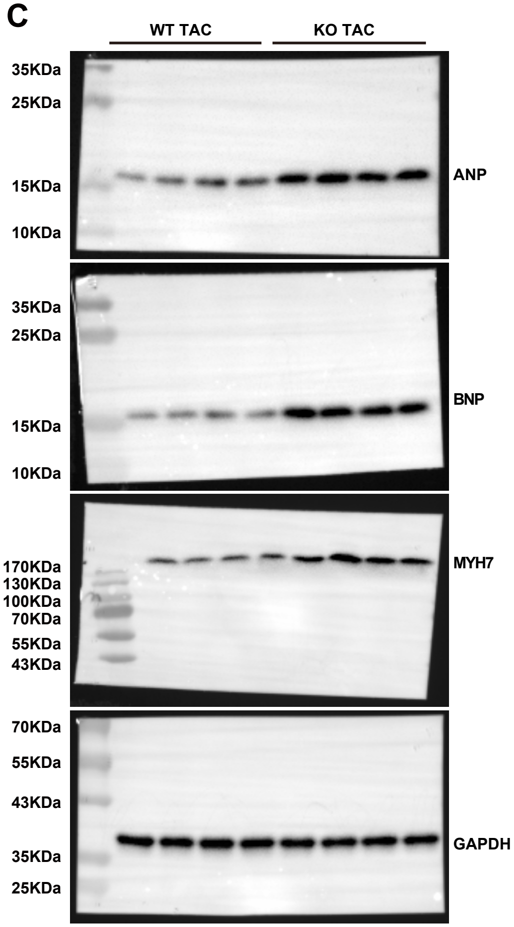

G

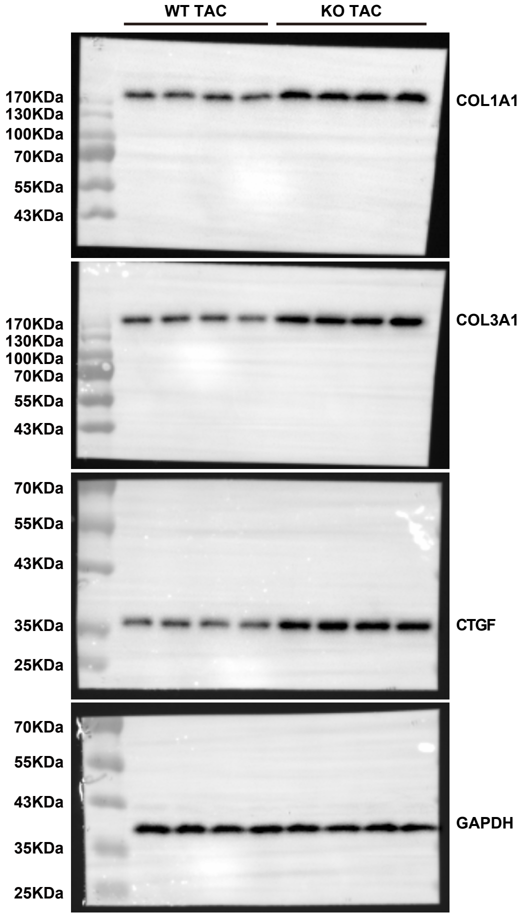

Figure4

A

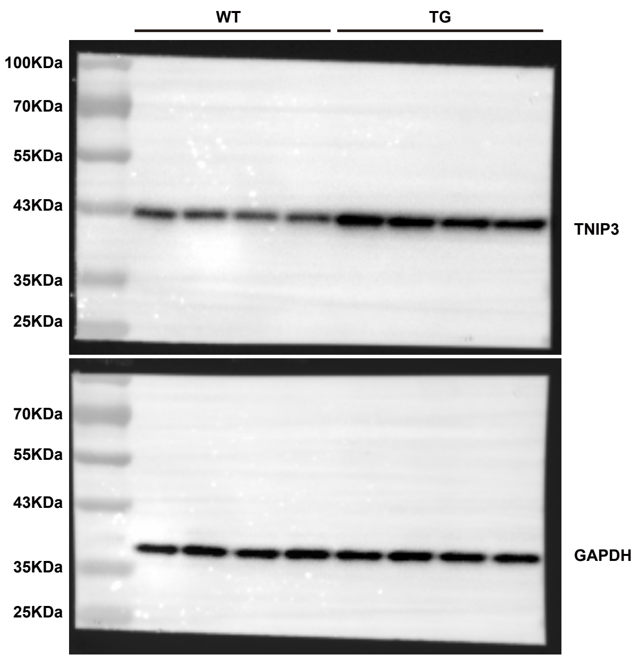
WT
TG
100KDa
70KDa
55KDa
43KDa
35KDa
25KDa
TNIP3
70KDa
55KDa
43KDa
35KDa
25KDa
GAPDH

F

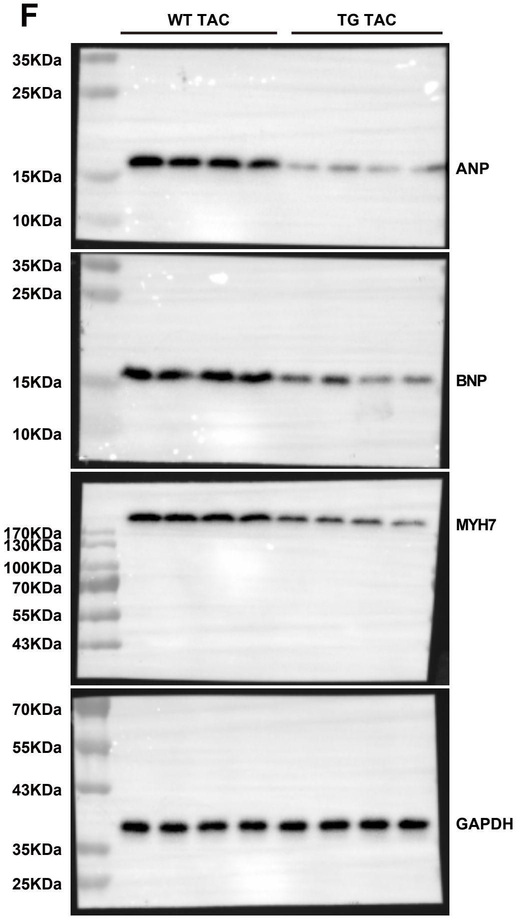
WT TAC
TG TAC
35KDa
25KDa
15KDa
10KDa
ANP
35KDa
25KDa
15KDa
10KDa
BNP
170KDa
130KDa
100KDa
70KDa
55KDa
43KDa
MYH7
70KDa
55KDa
43KDa
35KDa
25KDa
GAPDH

G

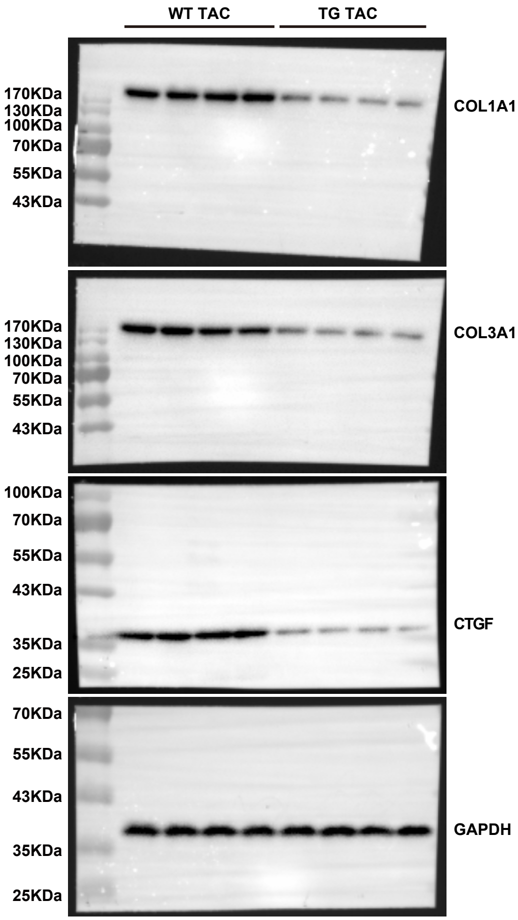
WT TAC
TG TAC
170KDa
130KDa
100KDa
70KDa
55KDa
43KDa
COL1A1
170KDa
130KDa
100KDa
70KDa
55KDa
43KDa
COL3A1
100KDa
70KDa
55KDa
43KDa
35KDa
25KDa
CTGF
70KDa
55KDa
43KDa
35KDa
25KDa
GAPDH

Figure5

A

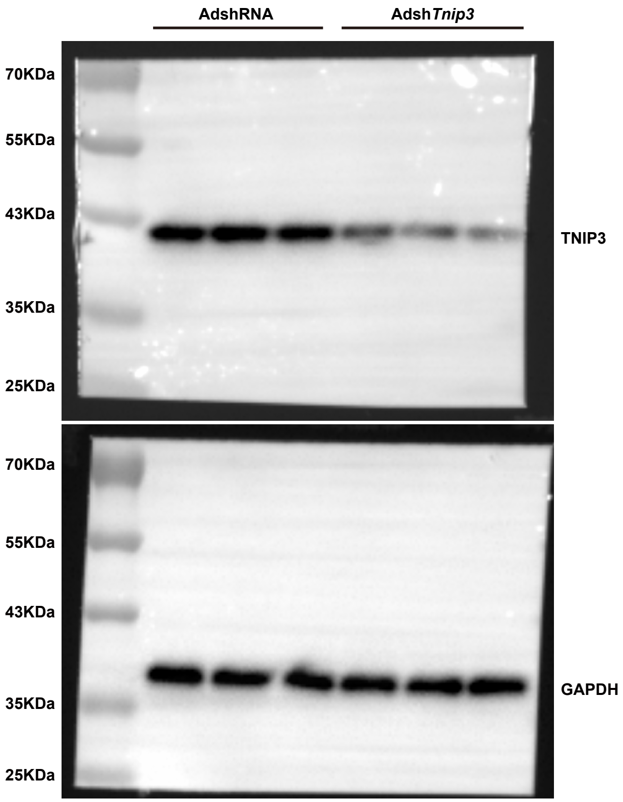
AdshRNA
Adsh*Tnip3*
70KDa
55KDa
43KDa
35KDa
25KDa
TNIP3
70KDa
55KDa
43KDa
35KDa
25KDa
GAPDH

D

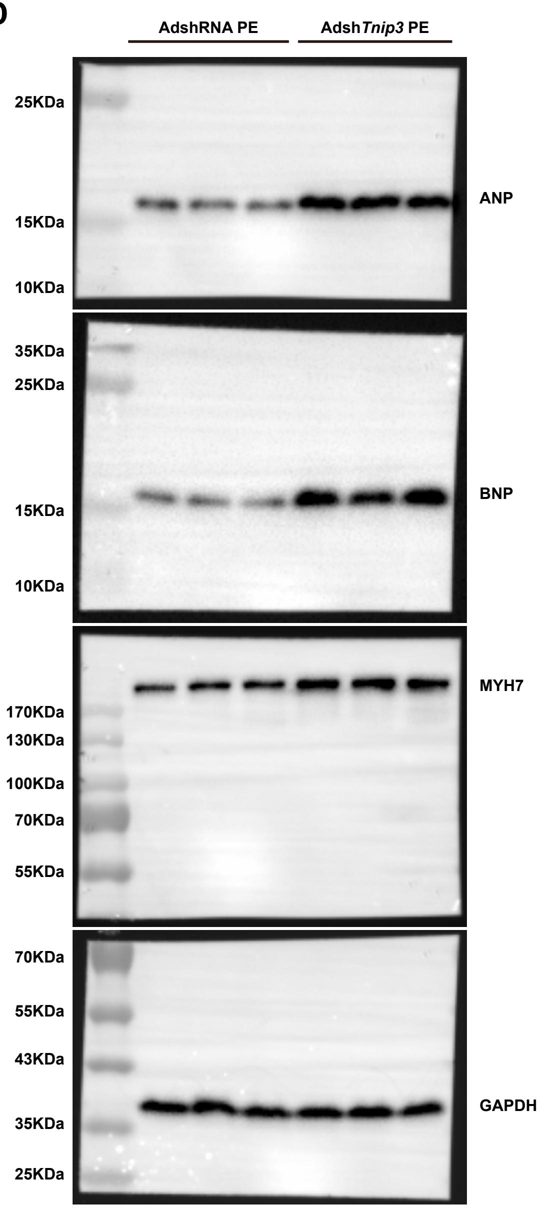
AdshRNA PE
Adsh*Tnip3* PE
25KDa
15KDa
10KDa
ANP
35KDa
25KDa
15KDa
10KDa
BNP
MYH7
170KDa
130KDa
100KDa
70KDa
55KDa
70KDa
55KDa
43KDa
35KDa
25KDa
GAPDH

Figure6

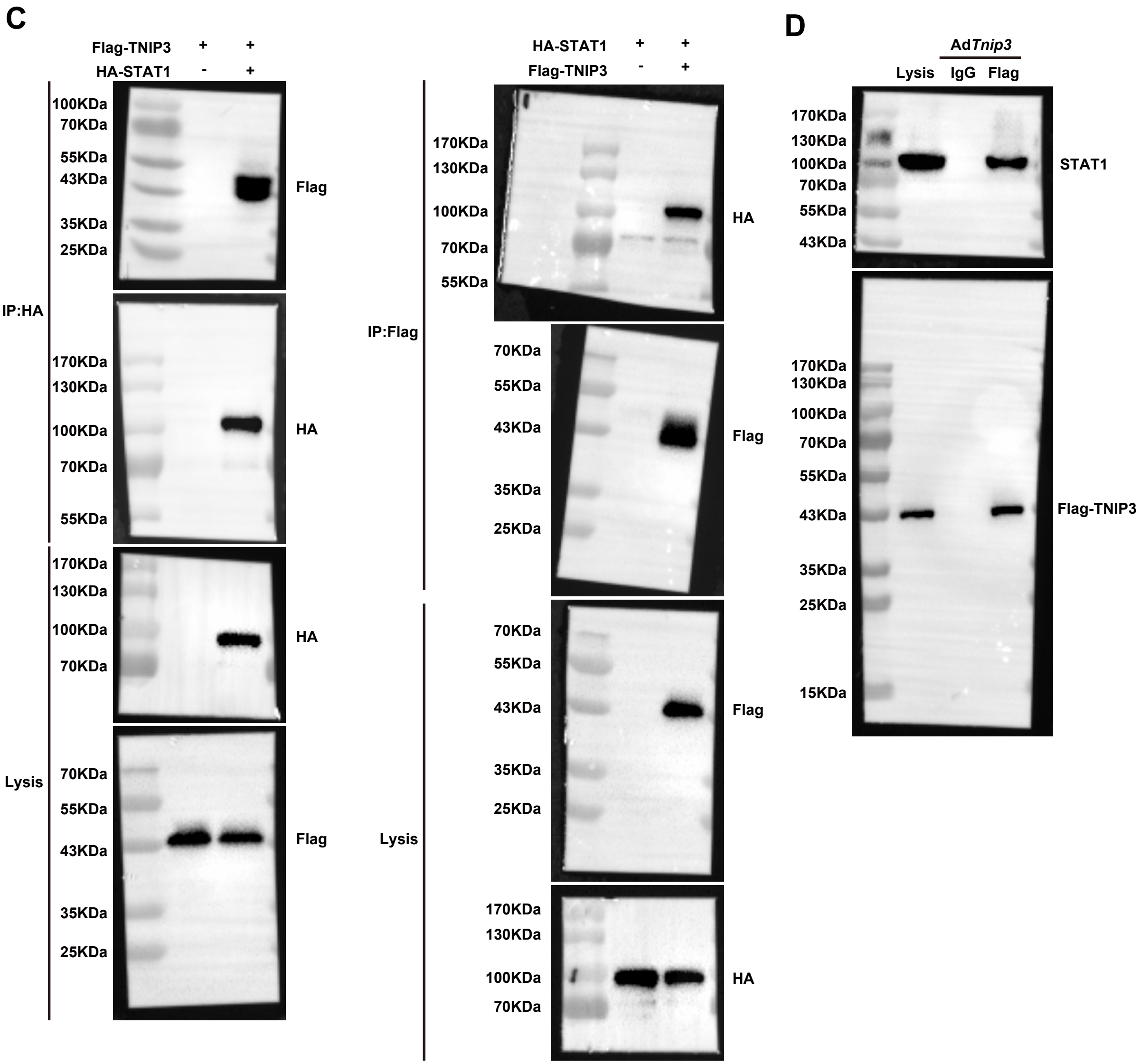
C
Flag-TNIP3 + +
HA-STAT1 - +
IP:HA
100KDa
70KDa
55KDa
43KDa
35KDa
25KDa
Flag
170KDa
130KDa
100KDa
70KDa
55KDa
HA
Lysis
170KDa
130KDa
100KDa
70KDa
HA
70KDa
55KDa
43KDa
35KDa
25KDa
Flag
HA-STAT1 + +
Flag-TNIP3 - +
IP:Flag
170KDa
130KDa
100KDa
70KDa
55KDa
HA
70KDa
55KDa
43KDa
35KDa
25KDa
Flag
Lysis
70KDa
55KDa
43KDa
35KDa
25KDa
Flag
170KDa
130KDa
100KDa
70KDa
HA
D
AdTnip3
Lysis
IgG
Flag
170KDa
130KDa
100KDa
70KDa
55KDa
43KDa
STAT1
170KDa
130KDa
100KDa
70KDa
55KDa
43KDa
35KDa
25KDa
15KDa
Flag-TNIP3

Figure6

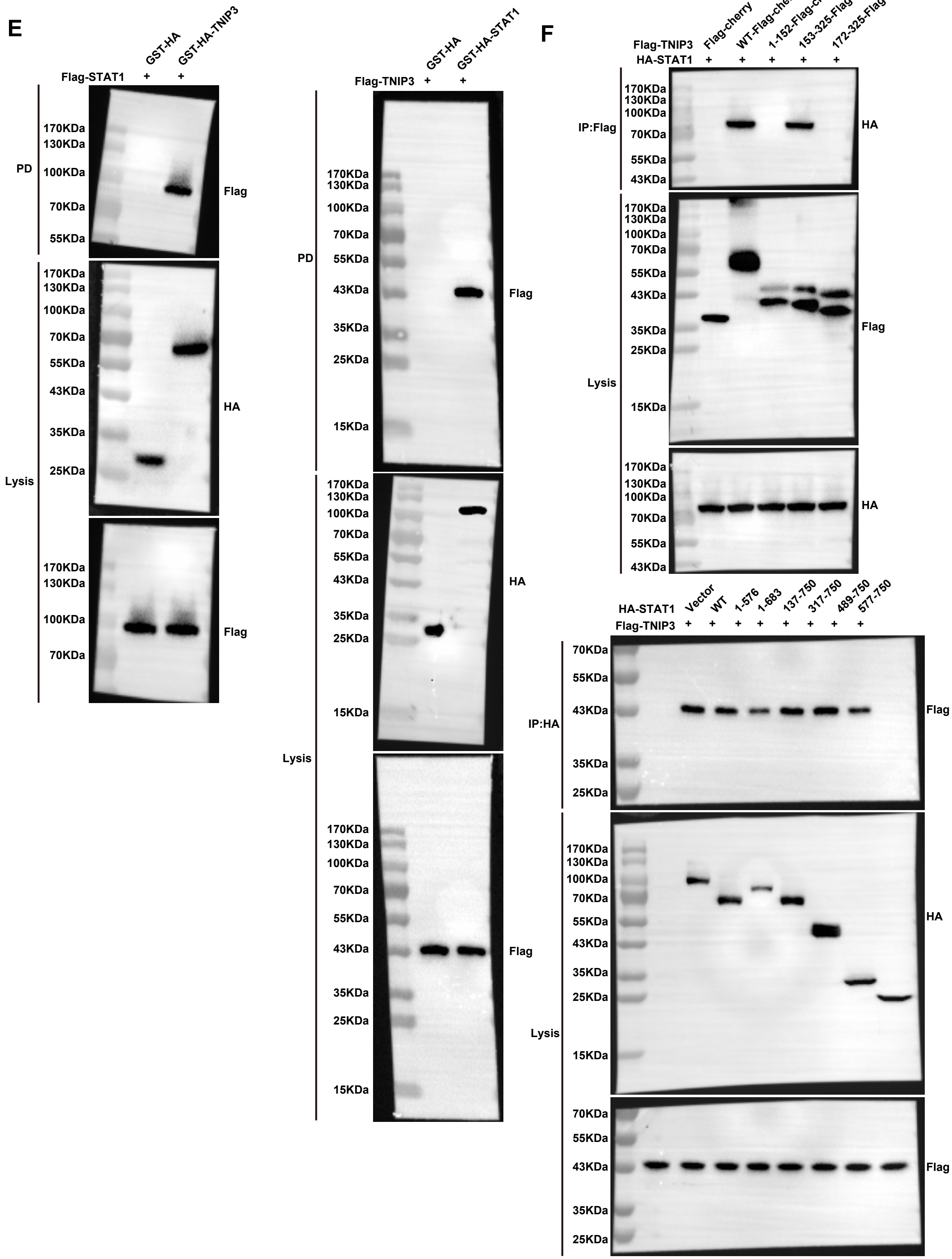
E
GST-HA
GST-HA-TNIP3
Flag-STAT1
PD
Lysis
Flag
HA
170KDa
130KDa
100KDa
70KDa
55KDa
43KDa
35KDa
25KDa
15KDa
GST-HA-STAT1
Flag-TNIP3
F
Flag-TNIP3
HA-STAT1
Flag-cherry
WT-Flag-cherry
1-152-Flag-cherry
153-325-Flag-cherry
172-325-Flag-cherry
IP:Flag
Vector
WT
1-576
1-683
137-750
317-750
489-750
577-750
IP:HA

Figure6

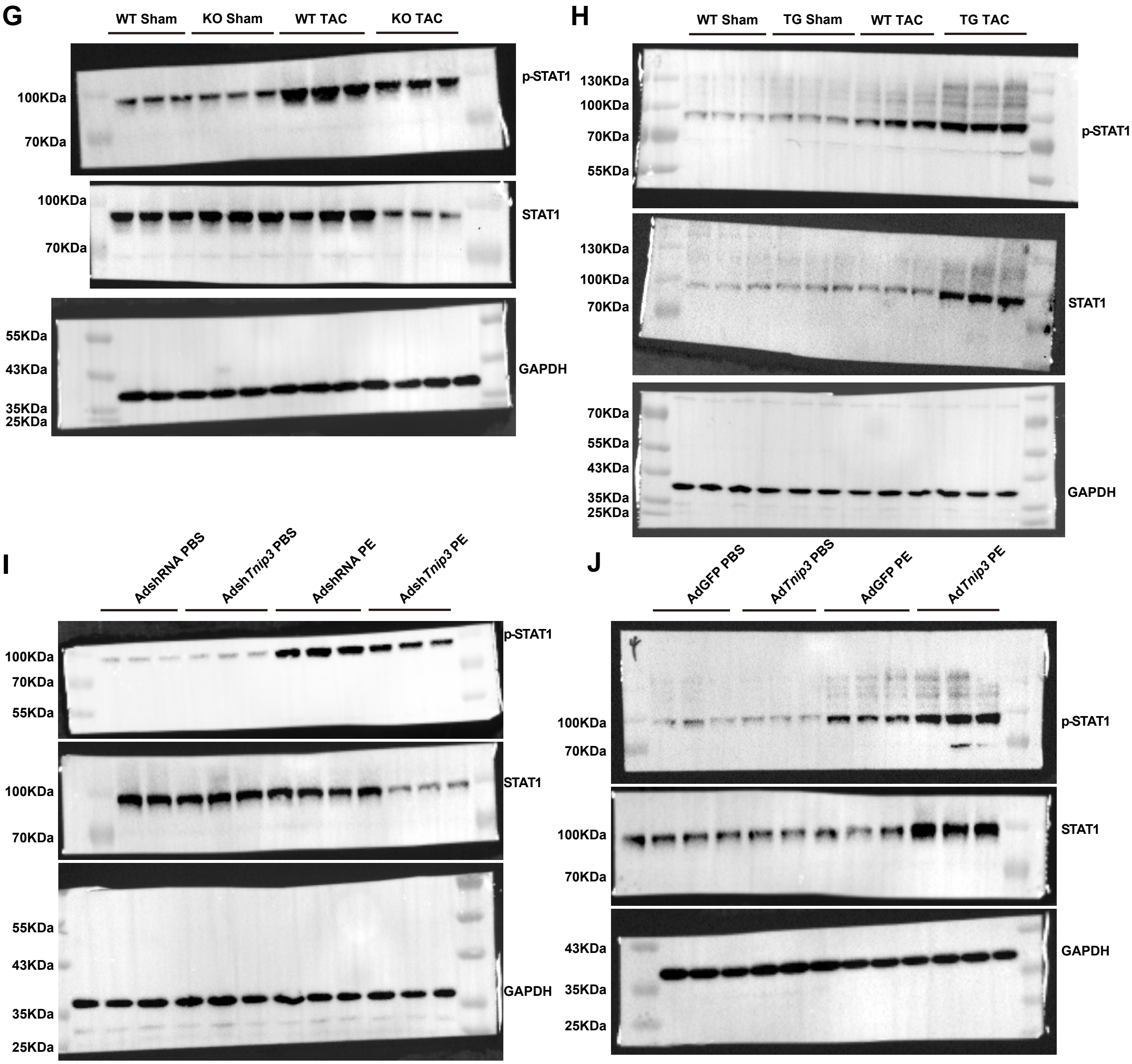
G
WT Sham
KO Sham
WT TAC
KO TAC
100KDa
70KDa
p-STAT1
100KDa
70KDa
STAT1
55KDa
43KDa
35KDa
25KDa
GAPDH
H
WT Sham
TG Sham
WT TAC
TG TAC
130KDa
100KDa
70KDa
55KDa
p-STAT1
130KDa
100KDa
70KDa
STAT1
70KDa
55KDa
43KDa
35KDa
25KDa
GAPDH
I
AdshRNA PBS
AdshTnip3 PBS
AdshRNA PE
AdshTnip3 PE
100KDa
70KDa
55KDa
p-STAT1
100KDa
70KDa
STAT1
55KDa
43KDa
35KDa
25KDa
GAPDH
J
AdGFP PBS
AdTnip3 PBS
AdGFP PE
AdTnip3 PE
100KDa
70KDa
p-STAT1
100KDa
70KDa
STAT1
43KDa
35KDa
25KDa
GAPDH

Figure7

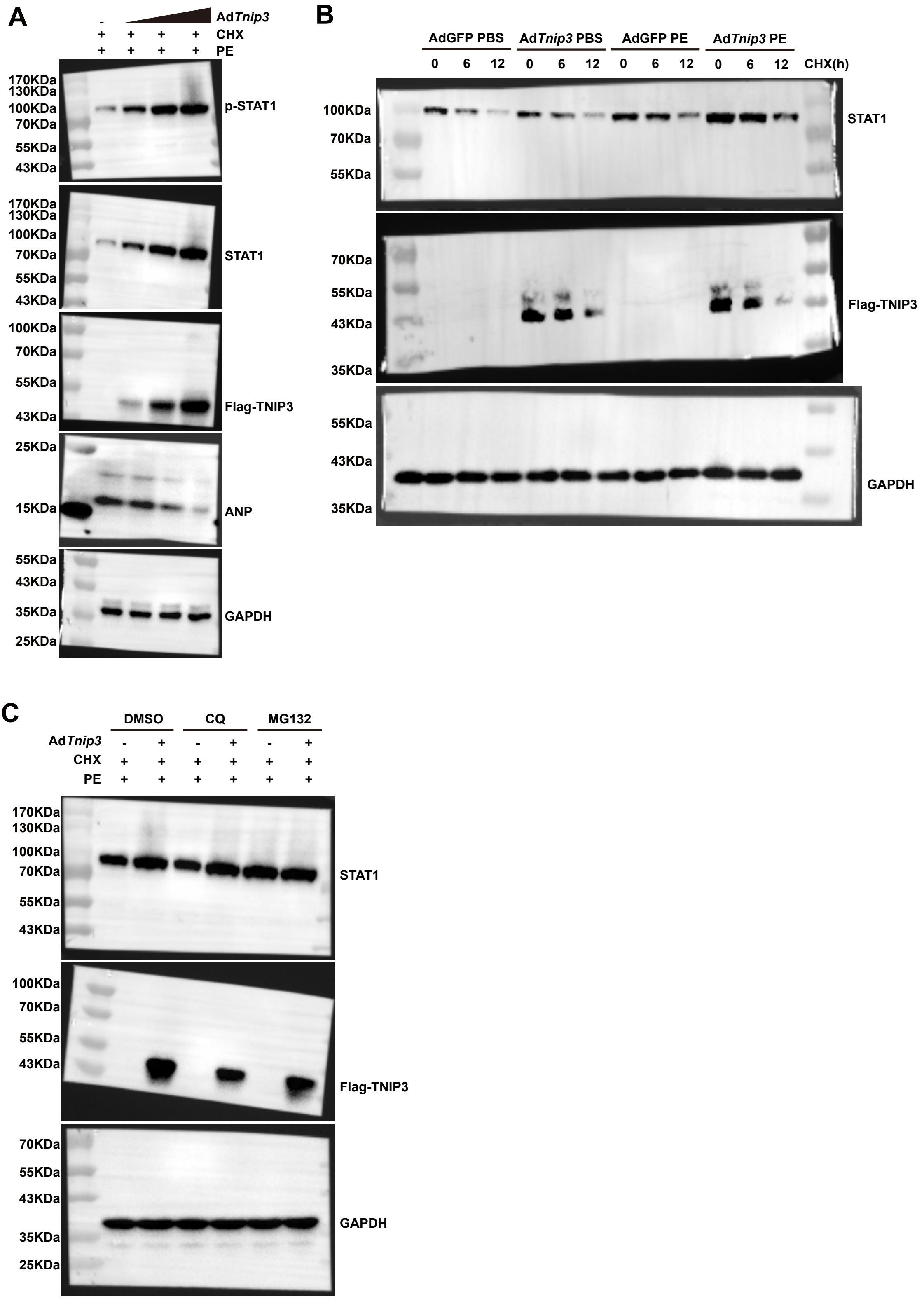
A
AdTnip3
CHX
PE
170KDa
130KDa
100KDa
70KDa
55KDa
43KDa
p-STAT1
STAT1
Flag-TNIP3
25KDa
15KDa
ANP
35KDa
GAPDH
B
AdGFP PBS
AdTnip3 PBS
AdGFP PE
AdTnip3 PE
0 6 12 0 6 12 0 6 12 0 6 12 CHX(h)
35KDa
C
DMSO
CQ
MG132

Figure7

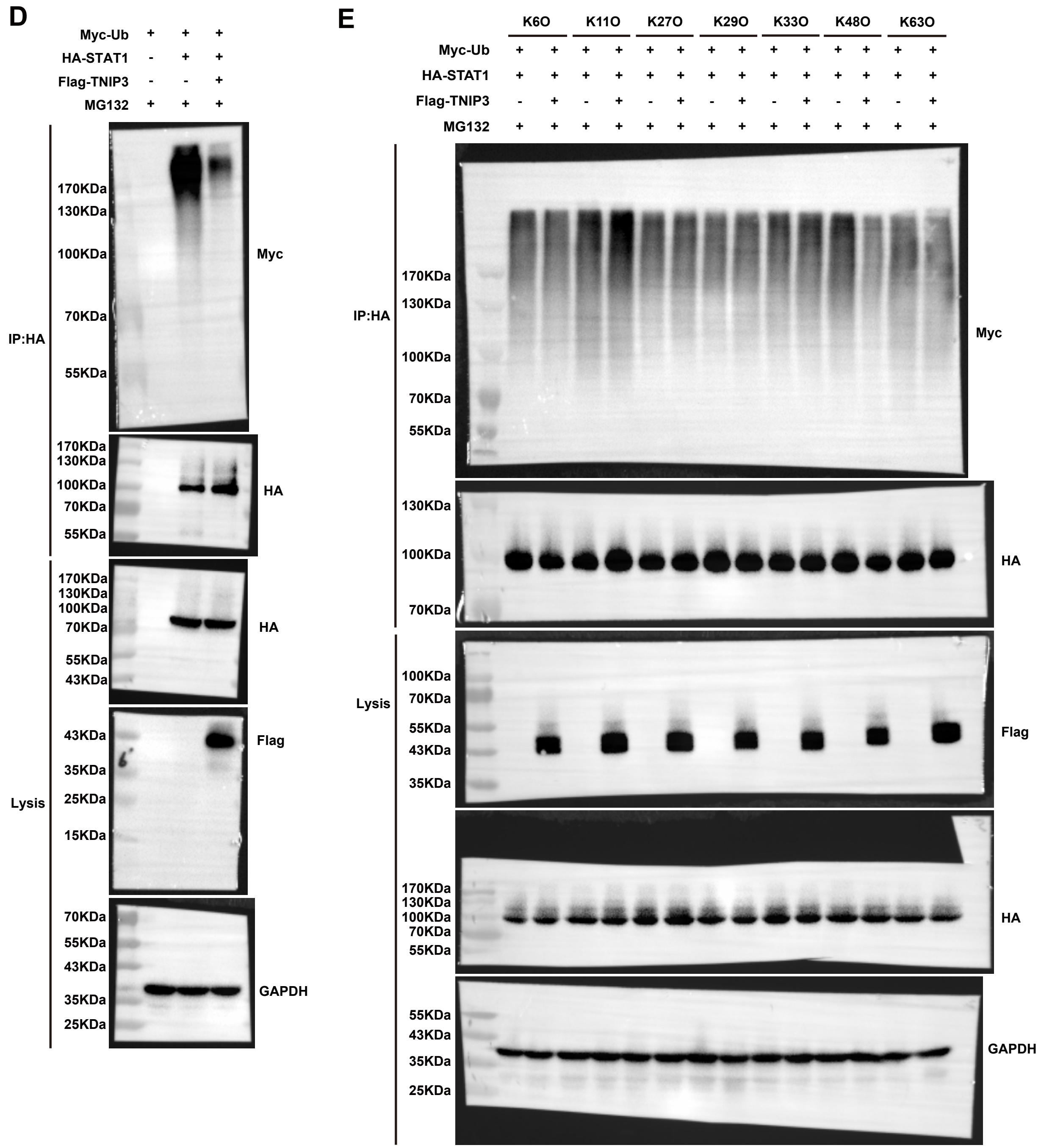

D
Myc-Ub + + +
HA-STAT1 - + +
Flag-TNIP3 - - +
MG132 + + +
IP:HA
170KDa
130KDa
100KDa
70KDa
55KDa
Myc
170KDa
130KDa
100KDa
70KDa
55KDa
HA
Lysis
170KDa
130KDa
100KDa
70KDa
55KDa
43KDa
HA
43KDa
35KDa
25KDa
15KDa
Flag
70KDa
55KDa
43KDa
35KDa
25KDa
GAPDH
E
K6O
K11O
K27O
K29O
K33O
K48O
K63O
Myc-Ub + + + + + + + + + + + + + +
HA-STAT1 + + + + + + + + + + + + + +
Flag-TNIP3 - + - + - + - + - + - + - +
MG132 + + + + + + + + + + + + + +
IP:HA
170KDa
130KDa
100KDa
70KDa
55KDa
Myc
130KDa
100KDa
70KDa
HA
Lysis
100KDa
70KDa
55KDa
43KDa
35KDa
Flag
170KDa
130KDa
100KDa
70KDa
55KDa
HA
55KDa
43KDa
35KDa
25KDa
GAPDH

Figure7

F

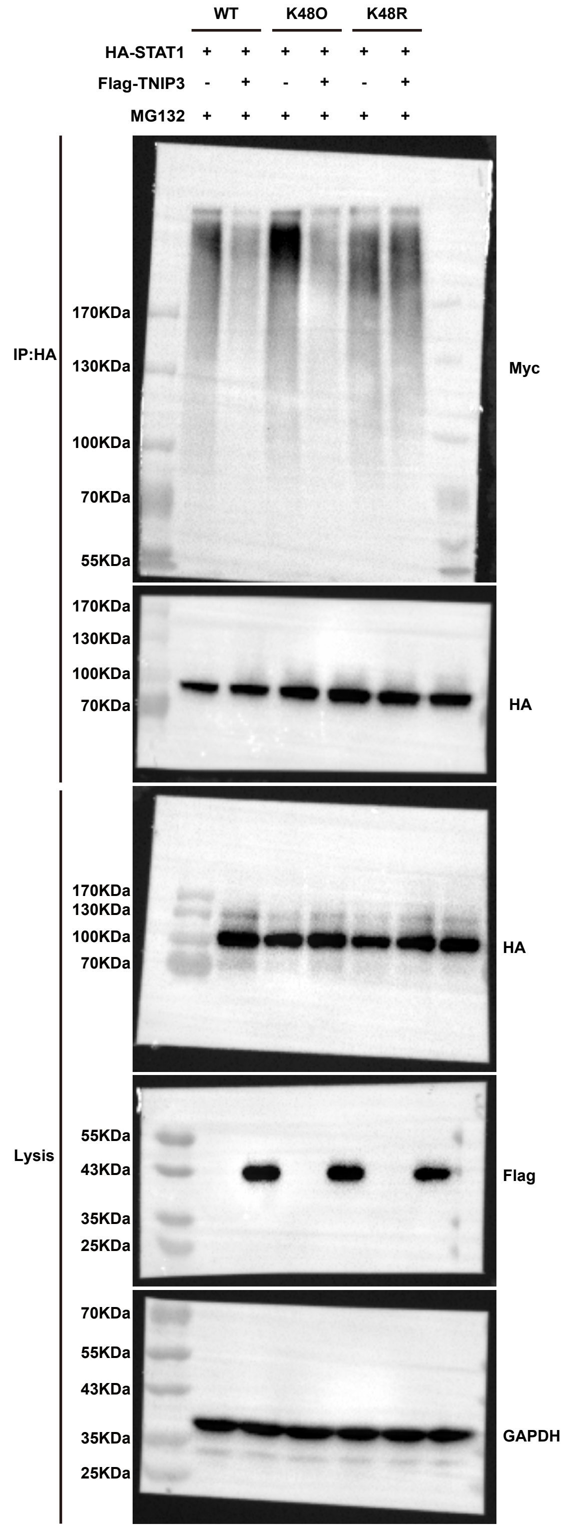

Figure8

A

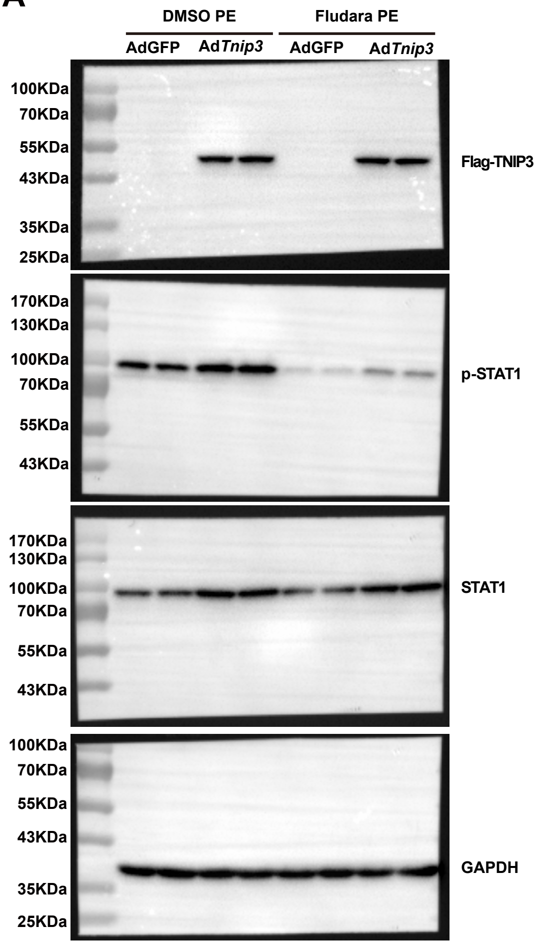

D

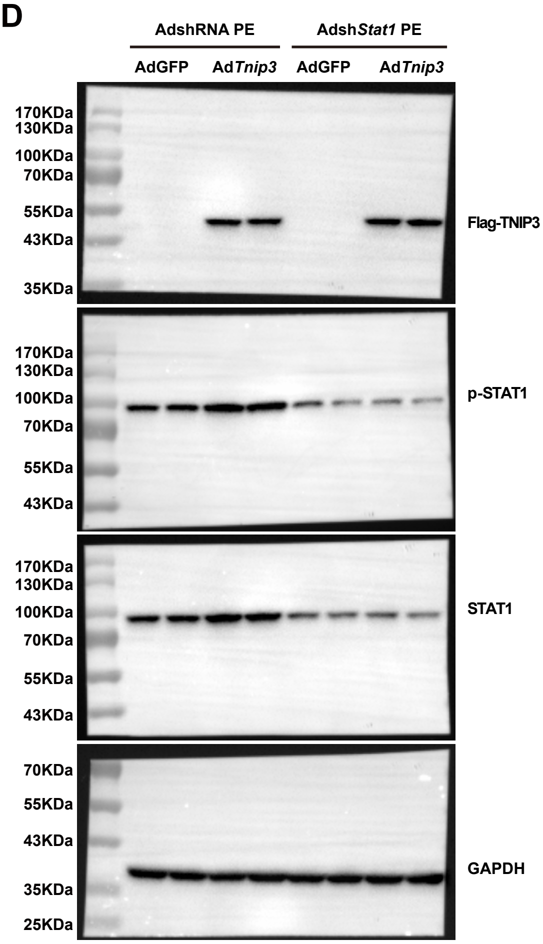

FigureS2

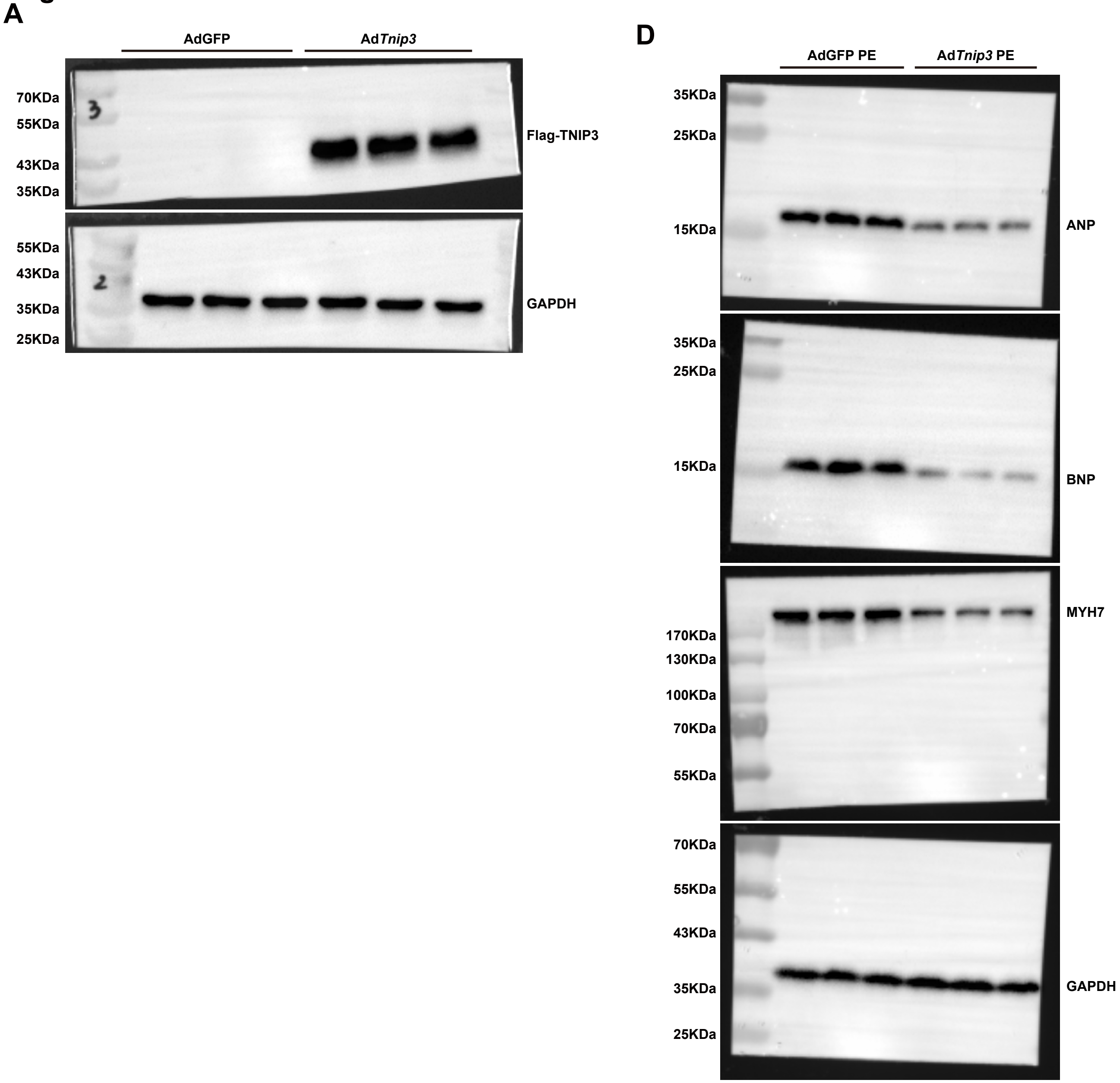
A
AdGFP
AdTnip3
70KDa
55KDa
43KDa
35KDa
Flag-TNIP3
55KDa
43KDa
35KDa
25KDa
GAPDH
D
AdGFP PE
AdTnip3 PE
35KDa
25KDa
15KDa
ANP
35KDa
25KDa
15KDa
BNP
MYH7
170KDa
130KDa
100KDa
70KDa
55KDa
70KDa
55KDa
43KDa
35KDa
25KDa
GAPDH

FigureS3

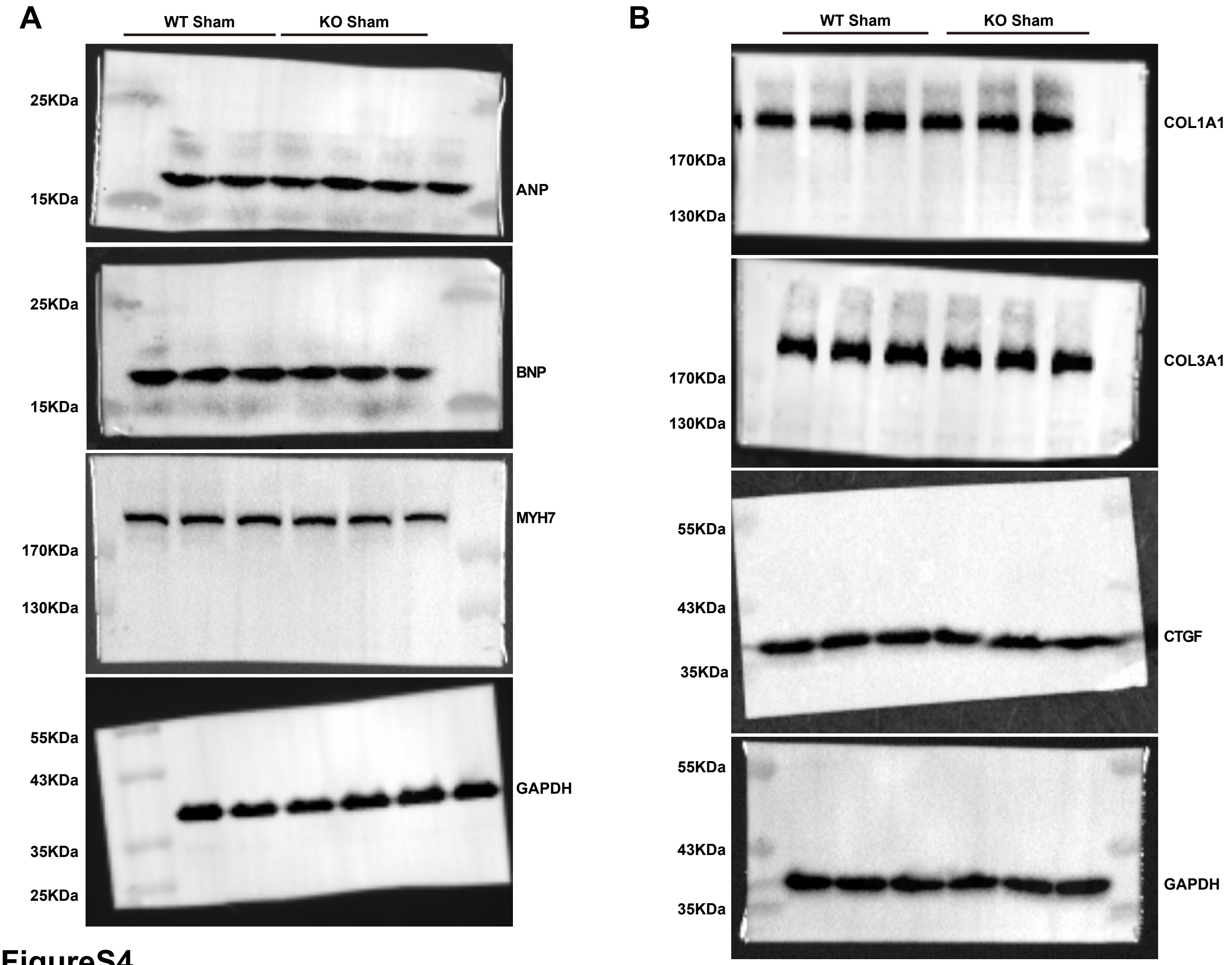

FigureS4

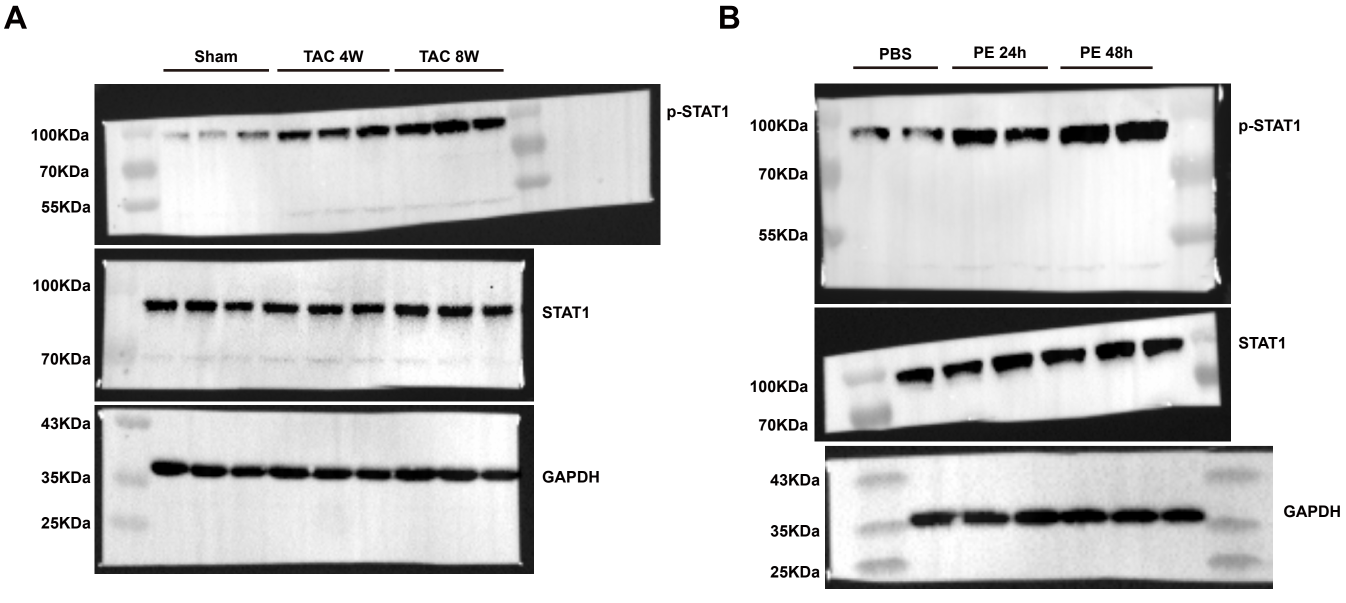

Supplement: Supplementary file 2 — Original western blots [file 41419_2024_6805_MOESM2_ESM.pdf]
